# Synthesis of novel phytol-derived γ-butyrolactones and evaluation of their biological activity

**DOI:** 10.1038/s41598-021-83736-6

**Published:** 2021-02-19

**Authors:** Anna Gliszczyńska, Katarzyna Dancewicz, Beata Gabryś, Marta Świtalska, Joanna Wietrzyk, Gabriela Maciejewska

**Affiliations:** 1 Department of Chemistry, Wroclaw University of Environmental and Life Sciences, Norwida 25, 50-375 Wrocław, Poland; 2 Department of Botany and Ecology, University of Zielona Góra, Szafrana 1, 65-516 Zielona Góra, Poland; 3 Department of Experimental Oncology, Ludwik Hirszfeld Institute of Immunology and Experimental Therapy, Polish Academy of Science, Weigla 12, 53-114 Wrocław, Poland; 4 Central Laboratory of Instrumental Analysis, Wrocław University of Science and Technology, Wybrzeże Wyspiańskiego 27, 50-370 Wrocław, Poland

**Keywords:** Biological techniques, Cancer, Cell biology, Chemical biology, Ecology

## Abstract

The synthesis of phytol-derived γ-butyrolactones as well as their evaluation for deterrent activity towards peach-potato aphid *Myzus persicae* and antiproliferative activity against four selected cancer cell lines are reported. Products were obtained in good yields (19–96%) and their structures were fully characterized by spectroscopic data (NMR, HRMS). Four synthesized δ-halo-γ-lactones (**4**–**7**) are new and have not been previously described in the literature. In the choice test phytol (**1**) appeared deterrent to *M. persicae*, whereas modifications of its structure did not cause the avoidance of the treated leaves by the aphids. In contrast, aphids were attracted to the leaves treated with the new *trans*-δ-chloro-γ-lactone (**6**). Electrical Penetration Graph (EPG) technique applied to explore the aphid probing and feeding activity revealed that neither phytol nor lactone **6** affected aphid probing and the consumption of phloem sap, which means that both phytol and the lactone **6** might have acted as postingestive modifiers of aphid behavior. The results of in vitro antitumor assays showed that obtained phytol derivatives exhibit cytotoxic activity against studied cancer cell lines (leukemia, lung and colon carcinoma and its doxorubicin resistant subline). Halolactones **4**–**6** were identified as the compounds, which arrest cell cycle of leukemia cells mainly in G2/M and S phases.

## Introduction

Mono-, di- and trisubstituted γ-butyrolactones are crucial structural elements of many biologically active natural and synthetic compounds. It has been estimated that almost 10% of natural products contain in their structure a γ-butyrolactone ring^1^. This type of molecules is produced by algae, sponges, fungi and liverworts^2–4^. Some of them have been also isolated from *Streptomyces* and *Hortonia* species^5,6^. γ-Butyrolactones determine the aroma and taste of fruits, vegetables and cheese^7–10^ and are valuable fragrance compounds of high-quality alcohol drinks such as wine, sherry, and whisky^11–13^. Among biological activities that γ-lactones show, to the fore comes extensively described in the literature their significant role which they play in the world of insects and the role of anticancer agents. This type of molecules is responsible for effective sexual signaling as sex attractant pheromones between different species of insect^14^, exhibits good antifeedant activity against insects^15^ and is also well-known as strong antitumor, antibiotic, antifungal and antiviral agents^16^.

Especially valuable γ-butyrolactones are those which structure is based on the isoprenoid skeleton. These lactones isolated from plants constitute one of the most numerous groups of antifeedants. However, their practical application is economically unjustified in the context of their low concentration in natural sources as well as high costs and difficulties with their isolation. From practical point of view more accessible are their synthetic analogs, like previously reported lactones synthesized from pulegone, limonene, myrtenol, pinene, damascone or farnesol^17–22^, for which it has been observed that their strong deterrent activity is mostly related to presence in their structure bromine or chlorine atom. For example, δ-halo-γ-lactones have been demonstrated as the most active lactone derivatives of damascone with strong antifeedant activity able to induce significant changes in the peach-potato aphid *Myzus persicae* behavior^20,23^. While *β*-damascone appeared a behaviorally inactive compound, the transformation of *β*-damascone into δ-bromo-γ-lactone caused frequent interruptions in the probing activity in peripheral plant tissues and a reduction in the ingestion of phloem sap^20^. Likewise, in the case of lactones with *p-*menthane system, the halolactones derived from piperitone had a negative effect on probing, feeding, and settling behavior of *M. persicae*, which was in contrast to the natural compound that showed weak attractant properties^24^. In some cases, like for racemic δ-halo-γ-lactone synthesized from farnesol it was shown that γ-butyrolactones are also able to inhibit the growth of cancer cell lines, A549 (human lung adenocarcinoma) and HL-60 (human promyelocytic leukemia)^16,23^.

Therefore, looking among isoprenoids for a biologically active natural carbon skeleton that could be the basis structure for developing its active lactone derivatives which can be useful as a new type of insecticides and cytostatic molecules we selected phytol (3,7,11,15-tetramethylhexadec-2-en-1-ol) (PYT) (**1**). Our interest in phytol (**1**) was inspired by its easy availability and reports on its broad spectrum of biological activity. PYT is an acyclic monounsaturated diterpene alcohol widely distributed in the plant world but can be also found in algae^25,26^, in bacteria^27,28^, in gut of the ruminant’s animals and their dairy products^29^. Biosynthesis of PYT goes mainly on the mevalonic acid (MVA) pathway^30^ or 2-C-methylerythritol-4-phosphate (MEP) pathway^28^. Phytol (**1**) plays an important role in the plant world being a structural element of chlorophyll responsible for its anchoring to the thylakoid membrane of chloroplast^31^. It is a cost-effective chemical widely used in the cosmetics industry as a fragrance component and well known as a non-mutagenic food additive. The world use of PYT reaches 0.1–1 metric ton per year and it’s a non-toxic effect for the mammalian was evaluated in the studies on albino mice and rats. The calculated lethal doses (LD_50_) of PYT for them are above 5 g and 10 g/kg, respectively^32^.

PYT is known to exhibit insecticidal activities against *S. zeamais* and some other insect species^33,34^. This diterpene alcohol was found as the major metabolite in the ethanolic extracts of *Petiveria alliaceae*, responsible for the insecticidal activity against eggs and nymphs of *Bemisia tabaci*^35^. Methew and Thoppil^36^ reported phytol to be an important constitute of essential oil of *Salvia splendens* and postulated its role in insecticidal activity against *Aedes albopictus* larvae. Many papers indicate also that phytol (**1**) exhibits cytotoxic potential against certain cancer cell lines: leukemia (MV4-11 and HeLa), breast (MCF-7), prostate (PC-3) and lungs (A-549)^37–39^ and is the substance promising for the treatment of cancer.

The low and well-characterized toxicity of PYT and its high tolerance by mammals makes this compound a strong candidate for the development of new and environmentally friendly insecticides and cytostatic agents. Therefore, the aim of this work was to obtain more biologically active halolactone derivatives of phytol (**1**) and evaluate them as the antifeedant and anticancer agents. We synthesized a series of novel PYT derivatives and evaluated their feeding-deterrent activity against the peach-potato aphid *Myzus persicae* (Sulz.) (Hemiptera: Aphididae) and antiproliferative activity against four selected cancer cell lines. The influence of novel PYT derivatives on aphid activities was assessed by monitoring the settling behavior of freely moving aphids in choice situation. We also monitored the individual phases of aphid probing and feeding in the no-choice situation using the Electrical Penetration Graph (EPG) technique which visualizes the movements of aphid mouthparts within individual plant tissues. The antiproliferative activity of synthesized phytol derivatives was evaluated against selected cancer cell lines (leukemia, lung and colon carcinoma and its doxorubicin resistant subline) whereas their molecular mechanism of action including of cell cycle, cell death evaluation and their influence on the activity of caspase 3/7 were studied towards human biphenotypic B myelomonocytic leukemia cell line MV4-11.

## Materials and methods

### Chemistry

#### General

Racemic mixture of *cis*/*trans* (35%:65%) isomers of phytol (**1**) (PYT) (97% purity), *N*-bromosuccinimide (NBS, 99% purity) and *N*-chlorosuccinimide (NCS, 98% purity) were purchased from Sigma-Aldrich Chemical Co. (St. Louis, MO, USA), while trimethylortoacetate was purchased from Fluka. Analytical grade acetic acid, sodium hydrogen carbonate, acetone, hexane, diethyl ether, tetrahydrofuran (THF), anhydrous magnesium sulfate, sodium chloride were purchased from Chempur (Poland).

Analytical Thin Layer Chromatography (TLC) was carried out on silica gel coated aluminium plates (DC-Alufolien Kieselgel 60 F254, Merck, Darmstadt, Germany) with a mixture of hexane, acetone and diethyl ether in various ratios as the developing systems. Compounds were visualized by spraying the plates with solution of 1% Ce(SO_4_)_2_ and 2% H_3_[P(Mo_3_O_10_)_4_] (2 g) in 10% H_2_SO_4_, followed by heating to 120–200 °C.

The products of chemical synthesis were purified by column chromatography on silica gel (Kieselgel 60, 230–400 mesh ASTM, 40–63 μm, Merck) using a mixture of hexane, acetone, and diethyl ether (in various ratios) as eluents.

Gas chromatography (GC) analysis was carried out on an Agilent Technologies 6890 N Network GC instrument (Santa Clara, CA, USA) equipped with autosampler, split injection (20:1) and FID detector using a DB-5HT column (Agilent, Santa Clara, USA) (polyimide-coated fused silica tubing, 30 m × 0.25 mm × 0.1 µm) with hydrogen as the carrier gas. Products of the chemical reactions were analysed using the following temperature programme: injector 250 °C, detector (FID) 250 °C, initial column temperature: 100 °C, 100–300 °C (rate 30 °C/min), final column temperature 300 °C (hold 2 min).

Nuclear magnetic resonance spectra ^1^H NMR, ^13^C NMR, DEPT 135, HSQC, ^1^H–^1^H COSY and NOESY were recorded in CDCl_3_ solutions with signals of residual solvent (δ_H_ = 7.26 δ_C_ = 77) on a Brüker Avance II 600 MHz (Brüker, Rheinstetten, Germany) spectrometer.

High-resolution mass spectra (HRMS) were recorded using electron spray ionization (ESI) technique on spectrometer Waters ESI-Q-TOF Premier XE (Waters Corp., Milford, MA, USA).

#### General procedure for the synthesis of compounds (2–7)

The preparation of ester **2** and acid **3** has been illustrated in detail in our previous work^39^, and so the synthesis method would not be listed here.

To a solution of acid **3** (7.8 mmol) in THF (30 mL) the *N*-bromosuccinimide (7.8 mmol) or *N*-chlorosuccinimide (7.8 mmol) was added. The mixture was stirred at room temperature for 48–96 h. When the substrate reacted completely (TLC, GC) the mixture was diluted with diethyl ether and washed with saturated NaHCO_3_ solution and brine. Organic layer of ether extract was separated and dired over anhydrous magnesium sulfate and evaporated on a rotary evaporator. New δ-halogeno-γ-lactones (**4**–**7**) were separated by silica gel column using for elution hexane/diethyl eter in gradient system. Bromo- and chlorolactonization afforded products with the following physical and spectral data presented below:

##### *trans-5-Bromomethyl-4-methyl-4-(4′,8′,12′-trimethyltridecyl)dihydrofuran-2-one (****4****)*

(25% yield); ^**1**^**H NMR** (600 MHz, CDCl_3_): δ 0.85 (four t, J = 6.4 Hz, 12H, CH_3_-4′, CH_3_-8′, (CH_3_)_2_–12′), 1.05–1.55 (m, 21H, CH_2_-1′, CH_2_-2′, CH_2_-3′, CH_2_-5′, CH_2_-6′, CH_2_-7′, CH_2_-9′, CH_2_-10′, CH_2_-11′, H-4′, H-8′, H-12′), 1.24 (s, 3H, CH_3_-4), 2.30 and 2.60 (two d, J = 17.2 Hz, 2H, CH_2_-3), 3.47 (dd, J = 11.3, 7.3 Hz, 1H, one of CH_2_-Br), 3.55 (dd, J = 11.3, 4.5 Hz, 1H, one of CH_2_-Br), 4.39 (dd, J = 7.2, 4.5 Hz, 1H, H-5); ^**13**^**C NMR** (150 MHz, CDCl_3_): δ 19.61, 19.69 (CH_3_)_2_–12′), 22.65, 22.75 (CH_3_-4′, CH_3_-8′), 24.50 (CH_3_-4), 29.21 (CH_2_-Br), 41.49 (CH_2_-3), 42.86 (C-4), 22.00, 24.47, 24.82, 34.08, 37.28, 37.41, 37.61, 37.70, 39.38 (CH_2_-1′, CH_2_-2′, CH_2_-3′, CH_2_-5′, CH_2_-6′, CH_2_-7′, CH_2_-9′, CH_2_-10′, CH_2_-11′), 28.00, 32.69, 32.80 (H-4′, H-8′, H-12′), 87.66 (H-5), 174.92 (C-2); **HRMS (ESI):** m/z calcd. for C_22_H_41_BrO_2_ [M + Na]^+^ 439.2188; found 439.2182.

##### *cis-5-Bromomethyl-4-methyl-4-(4′,8′,12′-trimethyltridecyl)dihydrofuran-2-one (****5****)*

(46% yield); ^**1**^**H NMR** (600 MHz, CDCl_3_): δ 0.85 (four t, J = 6.4 Hz, 12H, CH_3_-4′, CH_3_-8′, (CH_3_)_2_–12′), 1.04–1.55 (m, 21H, CH_2_-1′, CH_2_-2′, CH_2_-3′, CH_2_-5′, CH_2_-6′, CH_2_-7′, CH_2_-9′, CH_2_-10′, CH_2_-11′, H-4′, H-8′, H-12′), 1.08 (s, 3H, CH_3_-4), 2.38 and 2.49 (two d, J = 17.2 Hz, 2H, CH_2_-3), 3.48 (m, 2H, one CH_2_-Br), 4.41 (dd, J = 7.5, 4.5 Hz, 1H, H-5); ^**13**^**C NMR** (150 MHz, CDCl_3_): δ 18.96 (CH_3_-4), 19.69, 19.76 (CH_3_)_2_–12′), 22.65, 22.75 (CH_3_-4′, CH_3_-8′), 29.17 (CH_2_-Br), 42.70 (CH_2_-3), 43.09 (C-4), 22.20, 24.46, 24.82, 37.28, 37.39, 37.41, 37.49, 39.38, 39.96 (CH_2_-1′, CH_2_-2′, CH_2_-3′, CH_2_-5′, CH_2_-6′, CH_2_-7′, CH_2_-9′, CH_2_-10′, CH_2_-11′), 28.00, 30.95, 32.72 (H-4′, H-8′, H-12′), 86.46 (H-5), 174.76 (C-2); **HRMS (ESI):** m/z calcd. for C_22_H_41_BrO_2_ [M + Na]^+^ 439.2188; found 439.2183.

##### *trans-5-Chloromethyl-4-methyl-4-(4′,8′,12′-trimethyltridecyl)dihydrofuran-2-one (****6****)*

(21% yield); ^**1**^**H NMR** (600 MHz, CDCl_3_): δ 0.84 (four t, J = 6.4 Hz, 12H, CH_3_-4′, CH_3_-8′, (CH_3_)_2_–12′), 1.03–1.54 (m, 21H, CH_2_-1′, CH_2_-2′, CH_2_-3′, CH_2_-5′, CH_2_-6′, CH_2_-7′, CH_2_-9′, CH_2_-10′, CH_2_-11′, H-4′, H-8′, H-12′), 1.23 (s, 3H, CH_3_-4), 2.23 and 2.60 (two d, J = 17.2 Hz, 2H, CH_2_-3), 3.67 (dd, J = 12.1, 6.1 Hz, 1H, one of CH_2_-Cl), 3.73 (dd, J = 12.1, 4.6 Hz, 1H, one of CH_2_-Cl), 4.33 (dd, J = 7.2, 4.6 Hz, 1H, H-5); ^**13**^**C NMR** (150 MHz, CDCl_3_): δ 19.61, 19.67 (CH_3_)_2_–12′), 22.65, 22.75 (CH_3_-4′, CH_3_-8′), 24.67 (CH_3_-4), 42.30 (CH_2_-Cl), 41.48 (CH_2_-3), 42.38 (C-4), 22.09, 24.46, 24.82, 34.21, 37.29, 37.39, 37.61, 37.70, 39.38 (CH_2_-1′, CH_2_-2′, CH_2_-3′, CH_2_-5′, CH_2_-6′, CH_2_-7′, CH_2_-9′, CH_2_-10′, CH_2_-11′), 28.00, 32.70, 32.80 (H-4′, H-8′, H-12′), 87.45 (H-5), 175.21 (C-2); **HRMS (ESI):** m/z calcd. for C_22_H_41_ClO_2_ [M + Na]^+^ 395.2693; found 395.2698.

##### *cis-5-chloromethyl-4-methyl-4-(4′,8′,12′-trimethyltridecyl)dihydrofuran-2-one (****7****)*

(39% yield); ^**1**^**H NMR** (600 MHz, CDCl_3_): δ 0.85 (four t, J = 6.6 Hz, 12H, CH_3_-4′, CH_3_-8′, (CH_3_)_2_–12′), 1.04–1.56 (m, 21H, CH_2_-1′, CH_2_-2′, CH_2_-3′, CH_2_-5′, CH_2_-6′, CH_2_-7′, CH_2_-9′, CH_2_-10′, CH_2_-11′, H-4′, H-8′, H-12′), 1.06 (s, 3H, CH_3_-4), 2.38 and 2.47 (two d, J = 17.2 Hz, 2H, CH_2_-3), 3.67 (m, 2H, one CH_2_-Cl), 4.35 (dd, J = 6.4, 4.9 Hz, 1H, H-5); ^**13**^**C NMR** (150 MHz, CDCl_3_): δ 19.06 (CH_3_-4), 19.69, 19.76 (CH_3_)_2_–12′), 22.65, 22.74 (CH_3_-4′, CH_3_-8′), 42.52 (CH_2_-Cl), 42.39 (CH_2_-3), 42.54 (C-4), 22.14, 24.46, 24.83, 37.28, 37.37, 37.41, 37.49, 39.38, 40.16 (CH_2_-1′, CH_2_-2′, CH_2_-3′, CH_2_-5′, CH_2_-6′, CH_2_-7′, CH_2_-9′, CH_2_-10′, CH_2_-11′), 28.00, 32.64, 32.80 (H-4′, H-8′, H-12′), 86.24 (H-5), 175.04 (C-2); **HRMS (ESI):** m/z calcd. for C_22_H_41_ClO_2_ [M + Na]^+^ 395.2693; found 395.2697.

### Deterrent activity of phytol and its derivatives

#### Aphids, plants and compound application

The peach potato aphids *Myzus persicae* (Sulzer) and the Chinese cabbage *Brassica rapa* subsp. *pekinensis* (Lour.) Hanelt were reared in laboratory at 20 °C, 65% r.h., and L16:8D photoperiod. One to seven days old apterous females of *M. persicae* and 3-week old plants with 4–5 fully developed leaves were used for experiments. *M. persicae* were obtained from the laboratory culture maintained at the Department of Botany and Ecology for many generations since 2000. All experiments were carried out under the same conditions of temperature, relative humidity, and photoperiod. The bioassays were started at 10–11.a.m. Each compound was dissolved in 70% ethanol to obtain the recommended 0.1% solution^40^. All compounds were applied on the adaxial and abaxial leaf surfaces by immersing a leaf in the ethanolic solution of a given compound for 30 s.^20^. Control leaves of similar size were immersed in 70% ethanol that was used as a solvent for the studied compounds. Experiments were performed 1 h after the compounds application to allow the evaporation of the solvent. Every plant and aphid were used only once.

#### Aphid settling (choice test)

This bioassay allows the study of aphid host preferences under semi-natural conditions^41^. In the present study, aphids were given free choice between control and treated excised leaves that were placed in a Petri dish. Aphids were placed in the dish equidistance from treated and untreated leaves, so that aphids could choose between treated (on one half of a Petri dish) and control leaves (on the other half of the dish). Aphids that settled, i.e. they did not move, and the position of their antennae indicated feeding, on each leaf were counted at 1 h, 2 h, and 24 h intervals after access to the leaf. Each experiment was replicated 8 times (n = 8 replicates, 20 viviparous apterous females/replicate). Aphids that were moving or not on any of the leaves were not counted.

### Behavioral responses of aphids *Myzus persicae* during probing and feeding (no-choice test)

Aphid probing and the phloem sap uptake by *M. persicae* was monitored using the technique of electronic registration of aphid probing in plant tissues, known as EPG (= Electrical Penetration Graph), that is frequently employed in insect–plant relationship studies considering insects with sucking-piercing mouthparts^42–44^. In this experimental set-up, aphid and plant are connected to electrodes and thus made parts of an electric circuit, which is completed when the aphid inserts its stylets into the plant. Weak voltage is supplied in the circuit, and all changing electric properties are recorded as EPG waveforms that can be correlated with aphid activities and stylet position in plant tissues^45,46^. The parameters describing aphid behaviour during probing and feeding, such as total time of probing, proportion of phloem patterns E1 and E2, number of probes, etc., are good indicators of plant suitability or interference of probing by chemical or physical factors in individual plant tissues^44–46^. In the present study, aphids were attached to a golden wire electrode with conductive silver paint (epgsystems. eu) and starved for 1 h prior to the experiment. Probing behaviour of 12 apterous females/studied compound and control was monitored for 8 h continuously with Giga-4 and Giga-8 DC EPG with 1 GΩ of input resistance recording equipment (EPG Systems, Wageningen, The Netherlands). Each aphid was given access to a freshly prepared plant and each aphid/plant combination was used only once. Various behavioural phases were labelled manually using the Stylet + software (www.epgsystems.eu). The following aphid behaviours were distinguished: no penetration (waveform 'np' – aphid stylets outside the plant), pathway phase—penetration of non-phloem tissues (waveforms 'ABC'), phloem phase (salivation into sieve elements, waveform ‘E1’ and ingestion of phloem sap, waveform ‘E2’), and xylem phase (ingestion of xylem sap, waveform 'G'). Waveform ‘G’ occurred rarely irrespective of the treatment. Therefore, in all calculations the xylem phase was added to the pathway phase and termed as probing in non-phloem tissues. The E1/E2 transition patterns were included in E2. Waveform patterns that were not terminated before the end of the experimental period (8 h) were not excluded from the calculations. The parameters derived from EPG recordings were analyzed according to their frequency and duration in configuration related to activities in peripheral and vascular tissues.

### Statistical analysis

The data of the choice-test were analyzed using Student’s t-test (STATISTICA 13.1. package). If aphids showed clear preference for the leaf treated with the tested compound (*p* < 0.05), the compound was described as having attractant properties. If aphids settled mainly on the control leaf (*p* < 0.05), the compound tested in the respective choice-test was stated a deterrent. From the data thus obtained the relative index of deterrence (DI) was calculated: DI = (C − T/C + T) where C was the number of aphids settled on control leaf, T was the number of aphids settled on the leaf treated with the tested compound. The value of DI ranged between 1 (ideal deterrent) and − 1 (ideal attractant)^41^.

All statistical calculations related to data of the no-choice test were performed using StatSoft, Inc. (2014) STATISTICA (data analysis software system), version 12, www.statsoft.com. EPG parameters describing aphid probing behaviour (Table 2) were calculated manually and individually for every aphid and the mean and standard errors were subsequently calculated using the EPG analysis Excel worksheet created for this study. The parameters derived from EPGs were analyzed according to their frequency and duration in configuration related to activities in peripheral and vascular tissues. The results were statistically analyzed using Mann–Whitney U-test where the values of EPG parameters recorded from aphids on treated plants were compared to control^41^.

### Antiproliferative activity of phytol and its derivatives

#### Cell lines

Human biphenotypic B myelomonocytic leukemia MV4-11, human colon cancer LoVo cell line and normal mouse fibroblast BALB/3T3 cells were obtained from American Type Culture Collection (Rockville, Maryland, USA), human lung carcinoma A549 cells were obtained from European Collection of Authenticated Cell Cultures (UK). All the cell lines are being maintained at the Hirszfeld Institute of Immunology and Experimental Therapy, PAS, Wroclaw, Poland and were cultured according the procedure described before^47^.

Cell lines were cultured according to the protocol described before^48^. MV4-11 cells were cultured in RPMI 1640 medium (Gibco, UK) with 1.0 mM sodium pyruvate, and 10% fetal bovine serum (FBS) (all from Sigma-Aldrich, Germany). A549, LoVo and LoVo/DX cells were cultured in RPMI 1640 + Opti-MEM (1:1) (both from Gibco, UK) supplemented with 5% fetal bovine serum, 1.0 mM sodium pyruvate (LoVo and LoVo/DX cells) (all from Sigma Aldrich Germany) and 0.1 μg/mL doxorubicin chloride (Accord) (only LoVo/DX cells). BALB/3T3 cells in Dulbecco medium (IIET, Poland) supplemented with 10% fetal bovine serum (Sigma-Aldrich, Germany). All culture media were supplemented with 2 mM l-glutamine (Sigma-Aldrich, Germany), 100 units/mL penicillin, and 100 µg/mL streptomycin (both from Polfa Tarchomin S.A., Poland). All cell lines were grown at 37 °C with 5% CO_2_ humidified atmosphere.

#### Determination of antiproliferative activity

The solutions of the compounds (50 mM) were prepared by dissolving the substances in DMSO (Sigma Aldrich, Germany). Then the tested compounds were diluted in culture medium to reach the final concentrations of 625, 125, 25 and 5 μM. Before adding of the tested compounds (24 h prior), the cells were plated in 96-well plates (Sarstedt, Germany) at a density of 1 × 10^4^ or 0.5 × 10^4^ (A549) cells per well. The assay was performed after 72 h of exposure to 625, 125, 25 and 5 μM of the tested agents. The in vitro cytotoxic effect of all agents was examined using the MTT (MV4-11) or SRB assay, described previously^47^. The results were calculated as an IC_50_ (inhibitory concentration 50%) the concentration of tested agent, which is cytotoxic for 50% of the cancer cells. IC values were calculated for each experiment separately and mean values ± SD are presented in Tables 3. Each compound in each concentration was tested in triplicate in a single experiment, which was repeated 3–5 times.

### Cell cycle analysis

Cell cycle analysis was carried out according to the procedure previously described^48^. The MV4-11 cells were seeded at the density of 1 × 10^5^ cells/well of culture medium on 24-well plates (Sarstedt, Germany) to the final volume of 2 mL and were exposed to the test compounds at concentrations 75 μM for 72 h. After incubation, the cells were collected and 1 × 10^6^ of cells were washed twice in cold PBS and fixed for 24 h in 70% ethanol at − 20 °C. Then the cells were washed twice in PBS and incubated with RNAse (8 μg/mL, Fermentas, Germany) at 37 °C for 1 h. The cells were stained for 30 min. with propidium iodide (50 μg/mL, Sigma Aldrich, Germany) at 4 °C and the cellular DNA content was analyzed by flow cytometry using BD LSRFortessa cytometer (BD Bioscience, San Jose, USA). Compounds at each concentration were tested at least three times independently. Obtained results were analyzed using Flowing software 2 (Cell Imaging Core, Turku Centre for Biotechnology, University of Turku Åbo Akademi University).

### Caspase-3/7 activity determination

Caspase-3/7 activity determination was performed according to the protocol previously described^48^. The MV4-11 cells were seeded at the density of 1 × 10^5^ cells/mL of culture medium on 24-well plates (Sarstedt, Germany) to the final volume of 2 mL. The cells were exposed to the test compounds at concentrations 75 μM or campthothecin (0.05 μg/mL) as a positive control, for 72 h. After 72 h of the incubation, the cells were collected and centrifuged (5 min., 4 °C, 250×*g*). Cells were suspended in 50 µL of ice-cold lysis buffer (50 mM HEPES, 10% (w/v) sucrose, 150 mM NaCl, 2 mM EDTA, 1% (v/v) Triton X-100, pH 7.3, IIET, Poland) and incubated 30 min. at 4 °C. After the incubation, 40 µL of each sample was transferred to a white, 96-well plate (Corning, USA) containing 160 µL of the reaction buffer (20 mM HEPES, 10% sucrose, 100 mM NaCl, 1 mM EDTA, 10 mM DTT, 0.02% Trition X-100, pH 7.3) (IIET, Wroclaw, Poland) with 9 µM Ac-DEVD-ACC fluorogenic substrate (λex = 360 nm, λem = 460 nm). The fluorescence increase correlated with the caspase-3/7 level was continuously recorded at 37 °C for 120 min using a Biotek Synergy H4 (Biokom, Warsaw, Poland). Compounds were tested in duplicates in single experiment and each experiment was repeated at least three times independently. Results were normalized to the number of cells in each well and are reported as mean relative caspase-3/7 activity compared to untreated control sample ± SD.

### Statistical analysis

Statistical analysis was performed in Statsoft Statistica 10. All datasets were analyzed using t-test. *p* Values lower than 0.05 were considered as statistically significant.

## Results and discussion

### Chemistry

**Figure 1:**
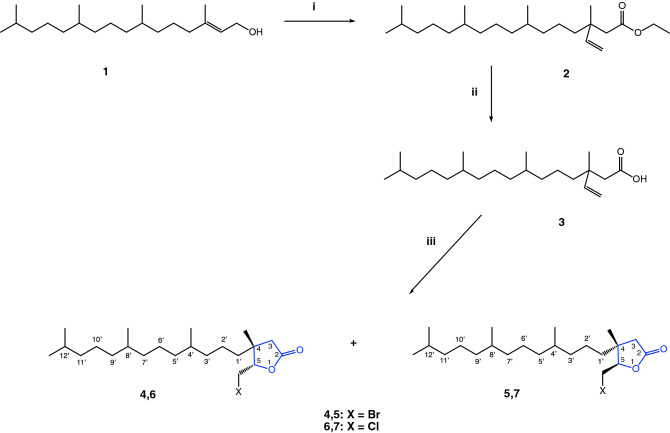
Synthesis of lactones derived from phytol (**1**). Reagents (i) CH_3_C(OEt)_3_, CH_3_COOH, 138 °C; (ii) 1. KOH, EtOH, 2. HCl; (iii) NBS/NCS, THF.

The synthesis of phytol-derived γ-butyrolactones is outlined in Fig. 1. The starting compound was commercially available mixture of *cis*/*trans* (35% : 65%) isomers of phytol (**1**) (PYT) (97% purity). This natural allylic alcohol (**1**) was subjected in the first step of the synthesis to the Johanson-Claisen rearrangement using triethylorthoacetate in the presence of a catalytic amount of propionic acid. The reaction afforded known ethyl 3,7,11,15-tetramethyl- 3-vinylhexadecanoate (**2**) in 86% yield, which was subsequently hydrolyzed in ethanolic KOH solution to the corresponding γ,δ-unsaturated acid (**3**) in 96% yield^39^. The acid **3** was next the substrate in the halolactonization reactions, which were carried out in tetrahydrofuran with *N*-bromosuccinimide or *N*-chlorosuccinimide, respectively and afforded four novel δ-halo-γ-lactones (**4**–**7**).

The bromolactonization afforded a mixture of two new products (**4**, **5**) in a ratio of 28% : 72% according to GC (after 2 days). We separated them using column chromatography and their structures were established based on spectroscopic methods. The products of cyclization were *trans*-δ-bromo-γ-lactone (**4**) (minor, 28%) and *cis*-δ-bromo-γ-lactone (**5**) (major, 72%). The lactones were obtained in 25 and 46% yield, respectively. The reaction of γ,δ-unsaturated acid (**3**) with *N*-chlorosuccinimide (NCS) was carried out to obtain chlorolactones (**6**, **7**). Using chloride as an electrophilic agent, we observed a similar situation as in the process of bromolactonization, but this time the reaction proceeded longer, 4 days. Two new δ-chloro-γ-lactones (**6**) and (**7**) were formed as products of cyclization. According to GC, the reaction mixture consisted 31% of *trans-*δ-chloro-γ-lactone (**6**) and 69% of *cis-*δ-chloro-γ-lactone (**7**), which were obtained in 21 and 39% yield, respectively. The structures of halolactones **4**–**7** were confirmed by ^1^H and ^13^C nuclear magnetic resonance (NMR) spectroscopy. Correlation spectroscopy (COSY), heteronuclear single quantum correlation spectroscopy (HSQC), nuclear overhauser effect spectroscopy (NOESY) and mass spectra (HRMS) were also applied for this purpose (all data are presented in the Experimental Section and all spectra are in Supplementary Materials).

^1^H NMR spectra of lactones **4**–**7** are similar to each other’s in terms of chemical shift values, shapes and multiplicities of signals. In all of them two characteristic doublets giving the typical AB-system at range 2.23–2.38 and 2.47–2.60 ppm with coupling constant *J* = 17.2 Hz from the CH_2_-3 protons are visible. At a lower frequency δ = 4.33–4.41 there are also doublet of doublets from the protons H-5. Signals from protons of five methyl groups were assigned on the basis of HMQC spectra. The most significant differences in chemical shifts values can be observed in the case of CH_2_-6 protons and it is an effect of the difference in the electronegativity of bromine and chlorine atoms, which are joined directly to the carbon atom C-6. With increasing electronegativity of the halogen atom shielding of methylene group protons decreases and the signals from these protons are shifted to the lower field (3.47 and 3.55 ppm in the case of bromolactones; 3.67 and 3.73 ppm in the case of chlorolactones). A considerable difference can be seen in the analysis of the position of the signal from the carbon atom C-6 in the ^13^C NMR spectra of bromo- and chlorolactones. The signals from C-6 carbon atom on bromolactones spectra are present at 29.21 and 29.17 ppm, respectively and at 42.30 and 42.52 ppm in the case of chlorolactones.

The spatial orientation of the halogenomethyl groups of synthesized bromo- and chlorolactones relative to the isoprenoid chain at carbon atom C-4 has been confirmed by spin–spin coupling through the space on the spectrum made in NOESY technique. Observed in the spectra of lactone **5** and **7** correlation signals confirmed the coupling of protons of halogenomethyl group with protons methyl group at C-4, indicating their close location in space (*cis* to each other). These correlations were not observed in the spectra of **4** and **5** made in NOESY technique. Formation of higher amounts of *trans*-lactones **5** and **7** where in their structure is *trans* location of halogenomethyl substituent and isoprenoid chain at C-4 can be explained by the preferential formation of bromonium/chloronium ion from the less shadowed side of double bound it means from the side of the methyl group, not a isoprenoid substituent. Carboxylate ion, according to the mechanism of halolactonization, formed a lactone ring, reacting from the side opposite to the bromine/chlorine atom.

### Biological studies

#### Behavioral response of *Myzus persicae* during settling: choice test

**Figure 2:**
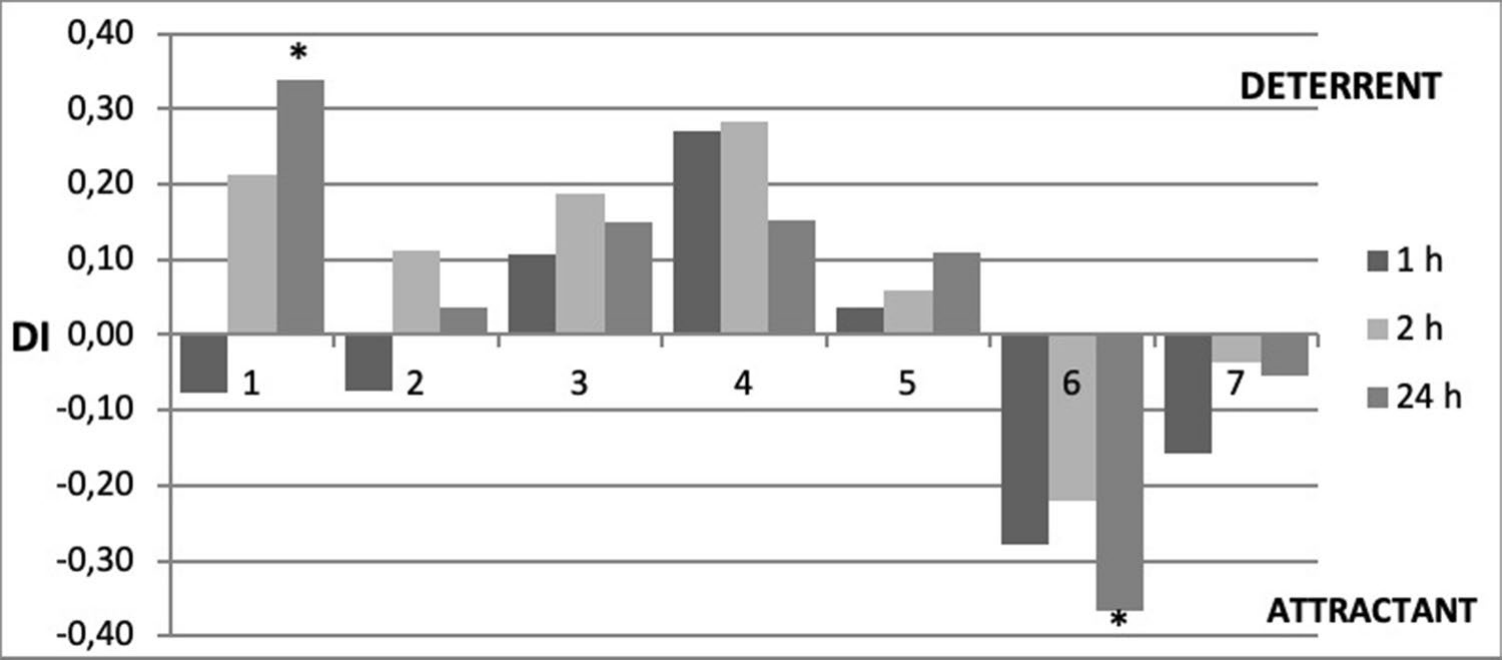
Settling success of *Myzus persicae* on *Brassica rapa* subsp. *pekinensis* exposed to phytol (**1**) and phytol-derived lactones (**2**–**7**). The same plant species was used to maintain the aphid stock culture. Relative index of deterrence (DI) after 1, 2, and 24 h. ‘*’Asterisks indicate statistically significant differences between the numbers of aphids on control and treated leaves at *p* < 0.05 (Student t-test).

The effect of phytol and its analogues was assessed by monitoring settling behavior of freely moving aphids. Aphids settle on a plant only when they accept it as a food source^49^. Therefore, the number of aphids that settle and feed on a given substrate is a good indicator of its suitability. Exogenously applied substances may alter plant suitability to aphids^18,20,50^. In the present work, we determined the settling success of the peach potato aphid after the application of phytol (**1**) and its newly obtained derivatives (**2–7**). The settling preferences of aphids depended on the compound and the time after the exposure of plants to the compound applied. The application of phytol (**1**) caused an initial acceptance of the treated leaves by *M. persicae* but from the second hour after application onwards, aphids tended to prefer the untreated to the treated leaves (Fig. 2, Table 1).

**Table 1:** Effect of phytol (**1**) and phytol-derived lactones (**2**–**7**) on settling of *Myzus persicae* on *Brassica rapa* subsp. *pekinensis*.

Compound		Mean number of aphids		
		1 h	2 h	24 h
**Phytol** (**1**)	Test	10.4 (± 1.3)	7.9 (± 1.0)	6.6 (± 0.9)
	Control	8.9 (± 1.0)	12.1 (± 1.0)	13.4 (± 0.9)
	*p*	0.3666	0.0099	0.0001
(**2**)	Test	10.8 (± 1.7)	8.9 (± 0.9)	9.6 (± 1.6)
	Control	9.3 (± 1.7)	11.1 (± 0.9)	10.4 (± 1.6)
	*p*	0.5447	0.1107	0.7404
(**3**)	Test	8.5 (± 2.1)	8.9 (± 1.0)	8.1 (± 1.3)
	Control	11.0 (± 2.1)	11.0 (± 1.0)	11.9 (± 1.0)
	*p*	0.1498	0.0562	0.3267
(**4**)	Test	2.9 (± 0.7)	2.4 (± 0.6)	4.9 (± 1.7)
	Control	5.0 (± 0.9)	4.3 (± 1.8)	6.6 (± 1.6)
	*p*	0.0950	0.3349	0.4767
(**5**)	Test	3.4 (± 1.0)	3.0 (± 1.1)	5.6 (± 1.4)
	Control	3.6 (± 0.9)	3.4 (± 0.9)	7.0 (± 1.9)
	*p*	0.8541	0.7890	0.5669
(**6**)	Test	5.8 (± 1.3)	4.5 (± 1.1)	10.0 (± 1.7)
	Control	3.3 (± 0.8)	2.9 (± 0.6)	4.6 (± 0.9)
	*p*	0.1244	0.2130	0.0089
(**7**)	Test	4.1 (± 1.0)	3.5 (± 0.7)	4.8 (± 0.9)
	Control	3.0 (± 1.2)	3.3 (± 1.4)	4.3 (± 1.2)
	*p*	0.4739	0.8781	0.7448

Numbers represent the number of aphids (means ± SD) settled on control and treated leaves; n = 8; significance determined at *p* < 0.05 according to Student t-test.

This indicates the deterrent properties of phytol (**1**) to *M. persicae*. Such switches in aphid responses in the course of time have previously been reported for other substances, such as piperitone-derived halolactones and hydroxylactones^24,51^. In contrast, aphids preferred the leaves treated with the new *trans-*δ-chloro-γ-lactone **6,** which indicates the attractant properties of this lactone to *M. persicae*. The aphid responses to phytol and lactone **6** were the strongest and statistically significant 24 h after exposure. The phytol derivatives **2–5** and **7** did not affect aphid settling behavior significantly. However, aphids showed a detectable tendency to avoid the leaves treated with the new γ,δ-unsaturated acid **3** and two new δ-bromo-γ-lactones **4** and **5** and prefer the leaves treated with the new *cis-*δ-chloro-γ-lactone **7** (Fig. 2, Table 1). Considering *M. persicae* response to phytol and phytol-derived lactones in the present study, it may be concluded that the freely moving aphids are less likely to settle on plants treated with phytol and more likely to settle on *trans-*δ-chloro-γ-lactone **6-**treated leaves than on untreated plants.

#### Behavioral responses of *Myzus persicae* during probing and feeding: no-choice test

The electronic monitoring of probing avtivities of *M. persicae* was applied to reveal the behavioral and physiological background of the activity of the studied compounds that was exposed in the observation of freely moving aphids in the aphid settling choice-test. The values of parameters derived from EPG recordings are reliable and accurate indicators of aphid behavioral responses to alteration in plant suitability due to exogenous application of xenobiotics^50,52^.

The typical behavior of *M. persicae* on control untreated plants consisted of non-probing (10% time of the 8-h experiment), probing in non-phloem tissues (35%), and probing in phloem tissues (55%). Sap ingestion occupied 98% of the phloem phase. Aphid probing activities were divided into 24.2 (± 4.8) probes on average and these probes were approximately 0.7 (± 0.3) hours long. *M. persicae* needed approximately 2.1 (± 0.4) hours and 14.1 (± 3.7) probes to reach phloem vessels and commence sap ingestion. 8.8 (± 2.5) of the probes before the first phloem phase were short eipdermal probes. The first contact with sieve elements (the first phloem phase) was 1.8 (± 0.7) hours long (Table 2). Stylet penetration in plant tissues was the main aphid activity throughout the duration of the experiment and phloem phase predominated among all probing activities (Fig. 2).

**Table 2:** Probing activities (EPG parameters) of *M. persicae* on *B. pekinensis* treated with phytol (**1**) and its derivatives (**2–7**) during 8-h EPG monitoring.

EPG parameters/compounds	Control	phytol (1)	(2)	(3)	(4)	(5)	(6)	(7)
General aspects of aphid probing behavior	n = 12	n = 12	n = 12	n = 12	n = 12	n = 12	n = 12	n = 12
Total duration of probing (C + G + E)^a^ (h)	7.2 ± 0.2	7.1 ± 0.2	7.3 ± 0.2	7.2 ± 0.2	7.4 ± 0.3	7.1 ± 0.4	6.6 ± 0.5	7.1 ± 0.5
Mean duration of probing (C + G + E)^a^ (h)	0.7 ± 0.3	0.3 ± 0.1	1.4 ± 0.6	1.6 ± 0.7	0.9 ± 0.3	0.5 ± 0.1	1.2 ± 0.7	1.0 ± 0.4
Number of probes	24.2 ± 4.8	41.4 ± 7.6	17.6 ± 4.1	25.3 ± 6.7	20.8 ± 5.2	31.6 ± 9.1	42.9 ± 10.4	18.7 ± 4.6
Total duration of pathway (C)^b^ (h)	2.7 ± 0.5	4.2 ± 0.4*	4.7 ± 0.8	2.9 ± 0.5	2.4 ± 0.6	2.8 ± 0.5	3.0 ± 0.5	2.5 ± 0.5
Total duration of phloem phase (E)^c^ (h)	4.4 ± 0.7	2.8 ± 0.5	2.4 ± 0.8	4.1 ± 0.8	4.8 ± 0.8	4.0 ± 0.7	3.5 ± 0.8	4.4 ± 0.8
Proportion of phloem phase in total probing^d^ (%)	58.9	38.4	32.0	52.6	62.9	54.0	46.7	56.9

Activities in non-phloem tissues	n = 11	n = 12	n = 10	n = 12	n = 12	n = 12	n = 10	n = 10
Time from first probe to first phloem phase^e^ (h)	2.1 ± 0.4	2.7 ± 0.6	2.9 ± 0.8	2.0 ± 0.5	2.7 ± 0.8	1.4 ± 0.3	3.0 ± 0.8	3.3 ± 0.8
Number of probes before first phloem phase^e^	14.1 ± 3.7	27.0 ± 5.3*	14.9 ± 4.1	15.7 ± 5.9	17.2 ± 4.8	10.3 ± 3.1	24.9 ± 9.0	15.6 ± 4.7
Number of probes < 3 min^e^	8.8 ± 2.5	18.8 ± 3.6*	9.1 ± 2.7	10.9 ± 4.4	8.5 ± 3.6	7.1 ± 2.8	20.3 ± 7.9	9.6 ± 3.6

Activities in sieve elements	n = 11	n = 12	n = 10	n = 12	n = 12	n = 12	n = 10	n = 10
Duration of first phloem phase^e^ (h)	1.8 ± 0.7	1.6 ± 0.5	2.0 ± 0.8	3.3 ± 0.9	3.0 ± 0.9	2.2 ± 0.8	2.0 ± 0.8	3.6 ± 0.8
Total duration of phloem sap ingestion E2 (h)	4.3 ± 0.7	2.7 ± 0.5	2.4 ± 0.8	3.9 ± 0.8	4.8 ± 0.8	4.0 ± 0.7	3.5 ± 0.8	4.4 ± 0.8
Total duration of phloem salivation E1 (min)	2.4 ± 0.6	8.4 ± 2.4*	2.4 ± 1.2	9.0 ± 6.0	1.8 ± 0.6	2.4 ± 0.6	3.0 ± 1.2	1.2 ± 0.6
Proportion of salivation in phloem phase^f^ (%)	2.0	18.3	7.9	8.4	1.5	3.8	9.7	0.4

Values represent means ± SE; n = number of replicates.

*Asterisks denote statistically significant differences in relation to control at *p* < 0.05; Mann–Whitney U-test.

^a^Probing phase: pathway phase + xylem phase + phloem phase (C + G + E).

^b^Pathway (probing in non-vascular tissues) with cell punctures (C).

^c^Phloem phase (E).

^d^Index calculated as: duration of phloem phase E/duration of probing phase C + G + E.

^e^First phloem phase: first salivation into sieve elements or first salivation and phloem sap ingestion (E1 or E1 + E2); all individuals were included in analysis; when an aphid did not show phloem phase, the time/number of probes to the first E1 or the first E1 + E2 was the time/number of probes until the end of the experiment.

^f^(duration of phloem salivation E1/duration of phloem phase E1 + E2)*100.

Generally, the behavior of *M. persicae* on plants treated with phytol was similar to the aphid behavior on control untreated plants. Aphids spent similar time on probing activities and the values of most EPG-derived parameters were similar to control (Table 2, Fig. 2). However, among the stylet probing activities, pathway activity predominated, which caused a slight decrease in the duration of the phloem phase. Aphids needed 2.7 (± 5.3) hours to reach phloem vessels. During that time, aphids inserted the stylets 27.0 (± 5.3) times, which was significantly more than in aphids on control plants. At the same time, the short epidermal probes were twice more numerous than on the control plants. The proportion of salivation during the phloem phase was four times higher than on control plants.

On plants treated with phytol derivatives **2–7**, no differences in aphid behavior were found in relation to control (Table 2, Fig. 4).

The values of parameters derived from EPG recordings are reliable and accurate indicators of aphid behavioral responses to alteration in plant suitability due to exogenous application of xenobiotics^20,24,41^.

**Figure 3:**
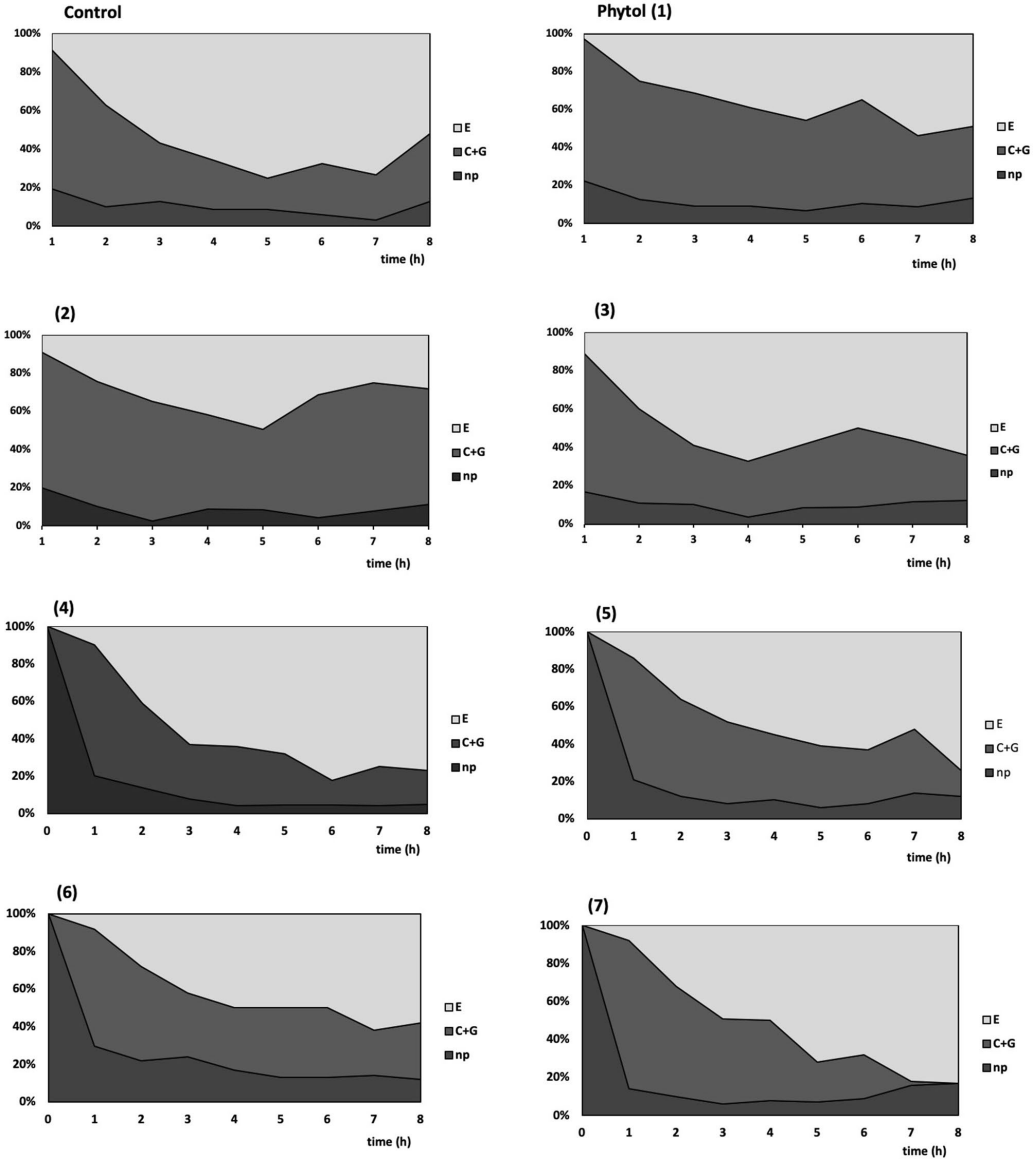
Sequential changes in EPG recorded probing and feeding behavior of *Myzus persicae* on *Brassica pekinensis* treated with phytol (**1**) and its derivatives (**2–7**), shown as the percentage of stylet activities during the 8-h experiment: np—no-probing; C + G—pathway phase: probing in non-phloem tissues mesophyll (C) and xylem (G); E—phloem phase: phloem salivation and phloem sap ingestion.

Insect feeding can be constrained at three levels: preingestive (immediate effect associated with host finding and host selection processes involving gustatory receptors), ingestive (related to food transport and production, release, and digestion by salivary enzymes), and postingestive (long-term effects involving various aspects of digestion and absorption of food)^53^. In the aphid settling choice test, we established that only phytol and the new trans-δ-chloro-γ-lactone **6** showed deterrent or attractant properties against *M. persicae*, respectively. The effects of these compounds on aphid behavior were revealed with a considerable delay in relation to the time when the aphids had access to the treated plants (Fig. 3). At the same time, we demonstrated that neither phytol nor any of the studied phytol-derivatives constrained aphid probing. The duration of the phloem sap ingestion activity was not altered on the treated plants, either, which means that aphids consumed similar amount of sap as on control plants during the experimental period. The rate of ingestion of the phloem sap is constant, therefore the amount of the consumed sap depends on the duration of ingestion^54^. Nevertheless, we may conclude that phytol had negative effect on *M. persicae* by changing the aphid behavior. Aphids walk away from the phytol-treated plants apparently after the sap has been consumed. We also found that aphids egested considerable amount of saliva during the phloem phase on phytol-treated plants, which was several times more than on control. The high proportion of salivation during the phloem phase suggests that phytol is not easily accepted by *M. persicae*. Watery saliva contains various enzymes associated with detoxification of plant allelochemicals, such as UPD-glucose transferases, polyphenol oxidases, and peroxidases^55^. Therefore, the long duration or frequent periods of watery salivation that interrupt sustained ingestion may indicate problems in sap acceptance^45^. It is very likely that phytol has postingestive deterrent effect on *M. persicae* behavior. In contrast to phytol, the lactone **6** did not cause excessive salivation during the phloem phase. At the same time, significantly more aphids settled on the new *trans*-δ-chloro-γ-lactone **6**-treated leaves 24 h after exposure. I our opinion, lactone **6** may be considered postingestive attractant. However, the hypotheses that refer to deterrent or attractant properties of phytol and lactone **6**, respectively need further testing before the practical application of these compounds is proposed.

### Antiproliferative activity of phytol and its derivatives

#### In vitro cytotoxic activities

The cytotoxic activities of the target compounds **2**–**7** in the in vitro model have been evaluated in MV4-11 (human biphenotypic B myelomonocytic leukemia) cell line using 3-(4,5-dimethylthiazol-2-yl)-2,5-diphenyl tetrazolium bromide (MTT) assay and A549 (human lung adenocarcinoma), LoVo (colon cancer) and LoVo/DX (doxorubicin resistant subline) cell lines using sulforhodamine B (SRB) assay. The toxicity of these compounds was also tested towards the normal mouse fibroblasts BALB/3T3. Cisplatin and doxorubicin were used as the positive controls and the results expressed as half-maximal inhibitory concentration (IC_50_) values are summarized in Table 3.

**Table 3:** Antiproliferative activity of phytol (**1**) and its synthesized derivatives against selected cancer cell lines and normal mice fibroblasts.

Compound	Cell lines/IC_50_ ± SD (μM)					
	MV4-11	A549	LoVo	LoVo/DX		BALB/3T3
	IC_50_ (μM)	IC_50_ (μM)	IC_50_ (μM)	IC_50_ (μM)	IR	IC_50_ (μM)
**Phytol (1)**	51.4 ± 3.6	64.1 ± 5.2	255.2 ± 46.5	70.8 ± 3.9	0.28	64.5 ± 12.9
**(2)**	n.a	n.a	n.a	n.a	–	n.a
**(3)**	39.28 ± 5.9	49.5 ± 2.9	59.5 ± 1.1	55.03 ± 1.5	0.92	145.1 ± 48.0
**(4)**	46.9 ± 11.7	n.a	n.a	306.3 ± 136.5	–	348.5 ± 27.6
**(5)**	52 ± 13.8	n.a	n.a	261.3 ± 104.5	–	424 ± 140.2
**(6)**	48.3 ± 19.6	378.4 ± 32.8	293.4 ± 33.1	187.5 ± 32.7	0.64	273 ± 16.4
**(7)**	110.5 ± 41.6	n.a	n.a	236.8 ± 52.9	-	n.a
**Cisplatin**	2.88 ± 0.83	3.97 ± 1.08	5.55 ± 0.12	3.45 ± 1.01	0.62	4.44 ± 2.36
**Doxorubicin**	-	-	0.12 ± 0.01	6.53 ± 0.93	–	–

n.a. no activity in concentration of 5, 25, 125, 625 μM. IC_50_—compound concentration leading to 50% inhibition of cell proliferation. Data are presented as mean ± standard deviation (SD) calculated using Prolab-3 system based on Cheburator 0.4 software^56^. IR was calculated according to the formula IR = (IC_50_ estimated against resistant cell line)/(IC_50_ estimated against non-resistant cell line); values range: 0 < IR < 2-indicate that the tested compound is able to overcome drug resistance; 2 < IR < 10—defines the moderate ability of the compound to overcome drug resistance; IR > 10—defines no influence on the drug resistance phenomenon.

Cell viability analysis indicates that synthesized derivatives show more pronounced cytotoxic activity than phytol (**1**). According to the values summarized in Table 3, it is shown that structural modification of PYT induces increase of the cytotoxic activity of obtained products and makes them more selective towards evaluated cell lines (Table 4). Among the tested cancer lines leukemia cells were the most sensitive to the inhibitory effect of all studied compounds (**1**–**7**). The phytol derivatives **2**–**7** were active towards leukemia at concentration range of 39.28–110.5 μM. In this group the highest activity was reported for 3,7,11,15-tetramethyl- 3-vinylhexadecanoic acid (**3**) and for *trans*-δ-bromo-γ-lactone (**4**) with IC_50_ = 39.28 and 46.9 μM, respectively. Considering the effect of phytol derivatives on the normal cells the potential application exhibit mainly δ-halo-γ-lactones (**4**–**6**) for which strong selectivity action towards cancer cells was observed. The obtained results of the antiproliferative effect of lactones **4**–**7** proved some correlation between their structures and cytotoxicity. While the differences in the activity of individual lactones containing bromine or chlorine atoms are almost not noticeable, in the case of orientation of the halogenomethyl group at carbon atom C-5 are clearly visible. *trans*-δ-Bromo-γ-lactone (**4**) and *trans-*δ-chloro-γ-lactone (**6**) exhibit higher activity than their *cis* analogues **5** and **7**.

**Table 4:** The selectivity index (SI) of tested compounds.

Compound	Cell lines/calculated selectivity index SI			
	MV4-11	A549	LoVo	LoVo/DX
**Phytol (1)**	1.25	1.01	0.25	0.91
**(2)**	–	–	–	–
**(3)**	3.69	2.93	2.44	2.64
**(4)**	7.43	–	–	1.14
**(5)**	8.15	–	–	1.62
**(6)**	5.65	0.72	0.93	1.46
**(7)**	–	–	–	–
**Cisplatin**	1.54	1.12	0.80	1.29

The SI index = IC_50_ for normal cell line (BALB/3T3)/ IC_50_ for respective cancerous cell line. A beneficial SI > 1.0 indicates a drug with efficacy against tumor cells greater than toxicity against normal cells.

In contrast to the cytotoxicity of phytol-derived products against MV4-11 cells the proliferation of human lung adenocarcinoma (A549) was inhibited only by acid **3** at the concentration 49.5 μM which was slightly lower than this appointed for phytol (**1**). The proliferation of colon cancer LoVo cells was also inhibited only by acid **3** and the established IC_50_ value 59.5 μM was 4 times lower than this observed for phytol (**1**) (IC_50_ 255.2 μM).

To evaluate agents activity against the MDR (multidrug resistance) phenotype cancer cell line LoVo together with its drug resistant subline LoVo/DX were tested and indexes of resistance (IR) were calculated. The IR value indicates how many times more a resistant subline is chemoresistant relative to its parental cell line. The data presented in Table 1 show that all compounds were more active against drug resistant LoVo/DX cells than against drug sensitive LoVo cells. Phytol (**1**) and compounds **3** and **6** were able to overcome drug resistance (IR < 2).

Many chemotherapeutics are toxic not only against malignant cells but also normal ones. Therefore, a very serious problem is the degree of toxicity of drugs depending on the doses required to achieve response in clinical trials. Selectivity of the cytotoxic activity of the phytol derivatives was determined by comparing the cytotoxic activity (IC_50_) of each compound against each cancerous cell line with that of the normal fibroblasts BALB/3T3 (Table 3). Results were expressed as the values of the Selectivity Index (SI). The selectivity indexes for new δ-halo-γ-lactones (**4**–**6**) against MV4-11 cells were much higher than for cisplatin. Phytol (**1**) also demonstrated lower selectivity towards cancer cells than its synthesized derivatives **3**–**7**. The 3,7,11,15-tetramethyl- 3-vinylhexadecanoic acid (**3**) had efficacy against all tested tumor cells greater than toxicity against normal cells.

#### Effect of phytol derivatives on cell cycle distribution of human leukemia cells MV4-11

**Figure 4:**
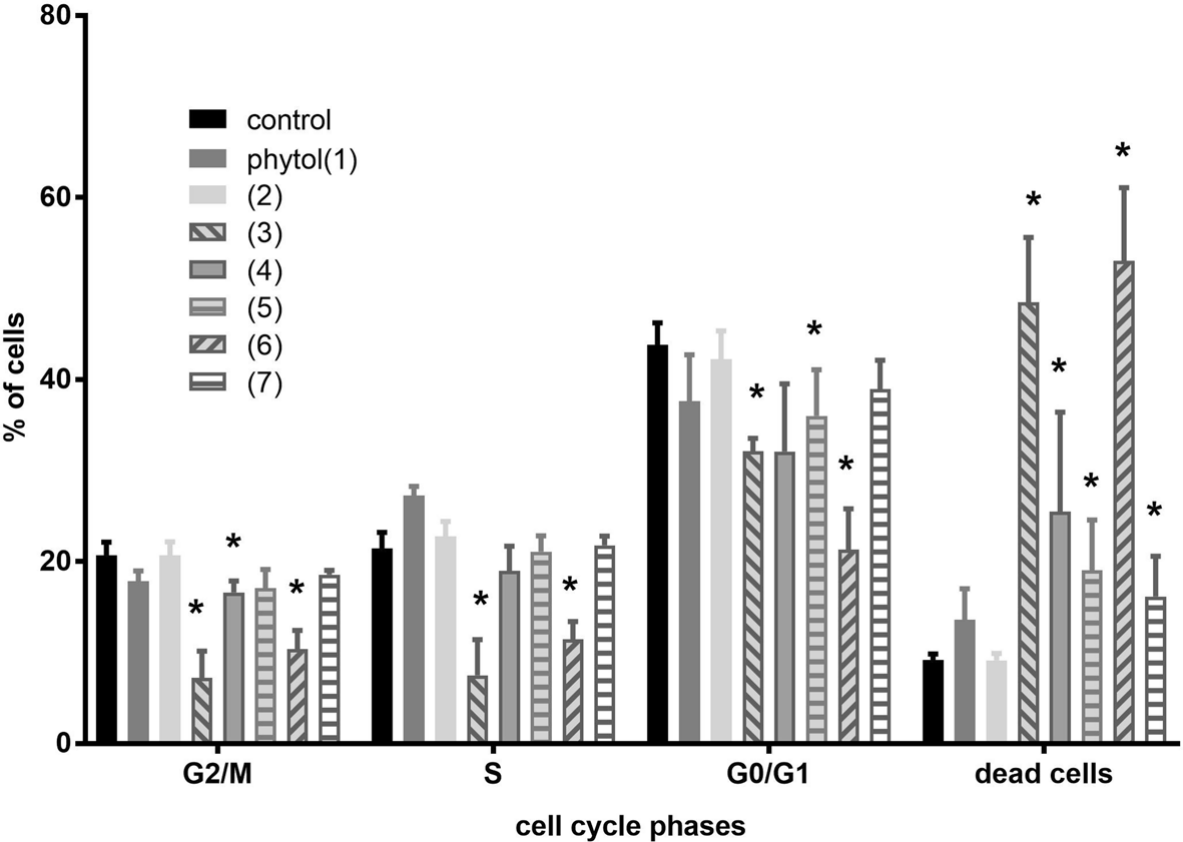
Cell cycle of MV4-11 cells induced by phytol derivatives at concentrations 75 µM.

**Figure 5:**
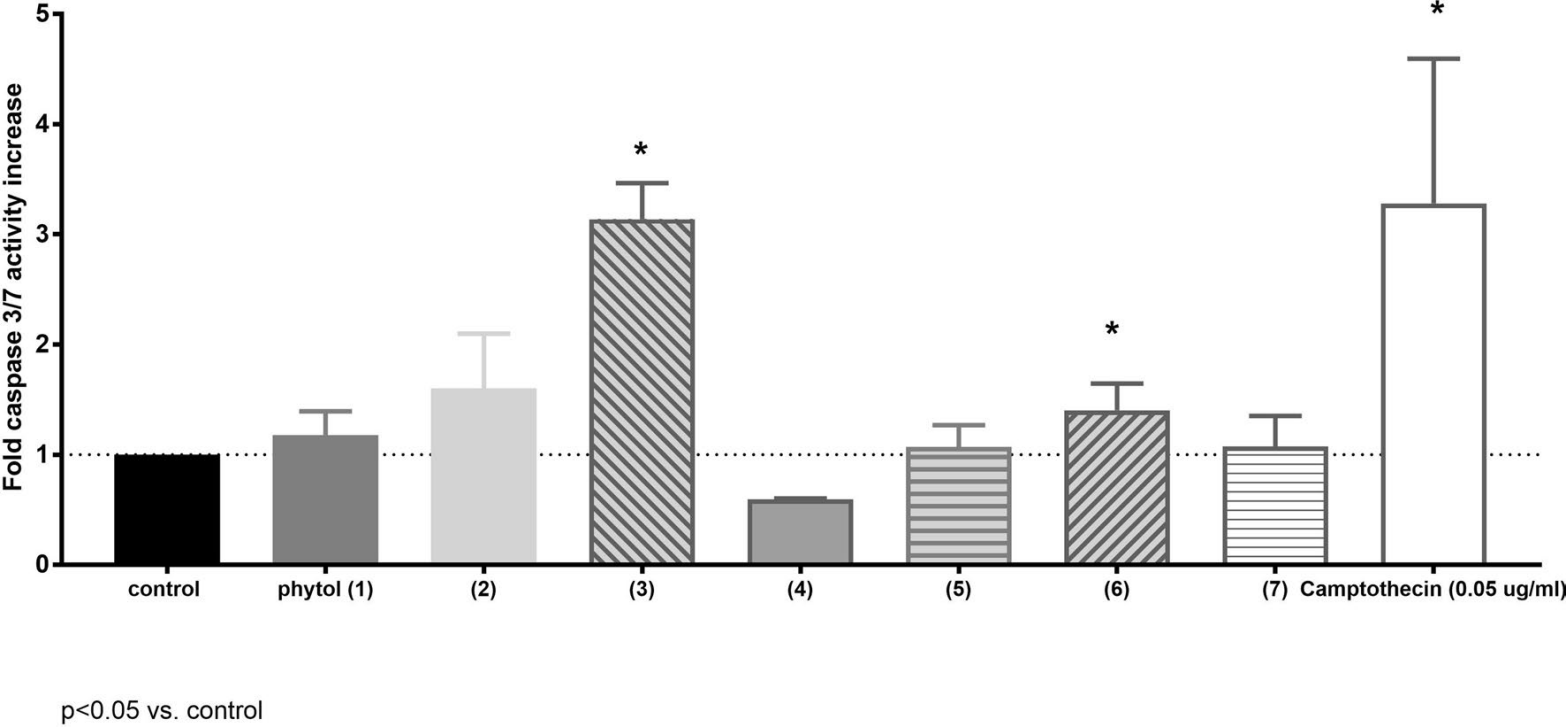
Caspase 3/7 activation in MV4-11 cells induced by phytol derivatives at concentrations 75 µM.

In the next step of the study the cell cycle of leukemia MV4-11 cells was analyzed after 72 h treatment of compounds in concentration 75 µM (Fig. 4). Compounds **3** and **6** induced death of 50% cells, which resulted lowering of cells number in G0/G1, S and G2/M phases (statistically significance in comparison to control cells, *p* < 0.05). This result is with agreement with result of analysis of caspase 3 activity (Fig. 5). Compounds **4**, **5** and **7** also induced death of 20–30% of cells.

#### Effect of phytol derivatives on activity caspase-3/7 expression in human leukemia cells MV4-11

One of the mechanisms involved in apoptosis is activation of caspase cascades. The main effector is caspase-3. To evaluate the expression of active caspas-3 we used peptidic substrates for caspase-3/7, Ac-Asp-Ph(F5)-Val-Asp-7-amino-4-carbamoylmethylcoumarin (Ac-DEVD-ACC). At the concentration of 75 μM the acid **3** increased activity of caspase-3/7 3-times in MV4-11 cells compared to the control and similar in comparison to reference drug camptothecin (0.05 μg/mL), whereas ester **2** and *trans-*δ-chloro-γ-lactone (**6**) increased activity of caspase about 1.5-times (results presented in Fig. 5). These three compounds exhibit potential as pro-apoptotic agents.

## Conclusion

In summary, a series of phytol-derived compounds were synthesized in good yields and their aphid behavior modifying activity and cytotoxic potency were evaluated.

The observation of freely moving aphids and the electronic monitoring of aphid probing in response to phytol and phytol-derived lactones demonstrated the deterrent potential of phytol and the attractant potential of the new *trans-*δ-chloro-γ-lactone **6.** Both phytol and the chlorolactone **6** acted as postingestive modifiers of aphid behavior. The phytol derivatives **2–5**, and **7** did not affect the feeding activity or settling behavior of *M. persicae*.

Phytol derivatives were also evaluated towards four human cancer cell lines (MV4-11, A549, LoVo, LoVo/DX) and normal mice fibroblasts (BALB/3T3). Obtained results confirmed that introduction of carboxy group and lactone ring into the structure of phytol increase its anticancer potential. Halolactones **4**–**6** were identified as the compounds, which arrest cell cycle of leukemia cells mainly in G2/M and S phases. We have found that the antiproliferative activity of individual lactones depends mainly on the orientation of halogenomethyl group at carbon atom C-5, whereas we did not observe bigger differences in the activity of bromo- and chlorolactones. However, further laboratory research in the area of the synthesis of more derivatives of phytol and broaden studies on their molecular mechanism of action are needed to be able to analyze the structure activity relationships. In the aspect of obtained results so far, the important fact is that calculated selectivity factors of almost all phytol derivatives were higher than those of cisplatin, what makes those products promising for further biological investigations. Intermediate acid **3** derived from phytol effectively inhibits the growth of all four types of tested cancer cells and is pro-apoptotic agent of leukemia cells that acts by activation of caspase-3/7 on the level comparable with camptothecin.

## Supplementary Information

Supplementary Information
