# Supplementary material for: Synthesis of novel phytol-derived γ-butyrolactones and evaluation of their biological activity
Source: Sci Rep. 2021 Feb 19;11:4262. doi: 10.1038/s41598-021-83736-6 (PMC7896091; doi:10.1038/s41598-021-83736-6)

*Supplementary Information*

# Synthesis of novel phytol-derived γ-butyrolactones and evaluation of their biological activity

Anna Gliszczyńska[1,*], Katarzyna Dancewicz[2], Beata Gabryś[2], Marta Świtalska[3], Joanna Wietrzyk[3], and Gabriela Mciejewska[4]

[1]Department of Chemistry, Wroclaw University of Environmental and Life Sciences, Wrocław, 50-375, Poland Norwida 25, 50-375, Wrocław, Poland

[2]Department of Botany and Ecology, University of Zielona Góra, Szafrana 1, 65-516 Zielona Góra, Poland

[3]Department of Experimental Oncology, Ludwik Hirszfeld Institute of Immunology and Experimental Therapy, Polish Academy of Science, Weigla 12, 53-114 Wrocław, Poland

[4]Central Laboratory of Instrumental Analysis, Wrocław University of Science and Technology, Wybrzeże Wyspiańskiego 27, 50-370, Wrocław, Poland

[*]anna.gliszczynska@wp.pl

## Content

Figure S1: $^{1}$H NMR spectrum of 4. .......... 4
Figure S2: $^{13}$C NMR spectrum of 4. .......... 5
Figure S3: $^{1}$H – $^{1}$H COSY spectrum of 4.......... 6
Figure S4: HSQC spectrum of 4. .......... 7
Figure S5: NOESY spectrum of 4. .......... 8
Figure S6: $^{1}$H NMR spectrum of 5. .......... 10
Figure S7: $^{13}$C NMR spectrum of 5. .......... 11
Figure S8: $^{1}$H – $^{1}$H COSY spectrum of 5.......... 12
Figure S9: HSQC spectrum of 5. .......... 13
Figure S10: NOESY spectrum of 5. .......... 14
Figure S11: $^{1}$H NMR spectrum of 6. .......... 16

Figure S12: $^{13}C$ NMR spectrum of 6. ........................................................................................ 17
Figure S13: $^{1}H$ – $^{1}H$ COSY spectrum of 6........................................................................................ 18
Figure S14: HSQC spectrum of 6. ........................................................................................ 19
Figure S15: NOESY spectrum of 6. ........................................................................................ 20
Figure S16: $^{1}H$ NMR spectrum of 7. ........................................................................................ 22
Figure S17: $^{13}C$ NMR spectrum of 7. ........................................................................................ 23
Figure S18: $^{1}H$ – $^{1}H$ COSY spectrum of 7........................................................................................ 24
Figure S19: HSQC spectrum of 7. ........................................................................................ 25
Figure S20: NOESY spectrum of 7. ........................................................................................ 26

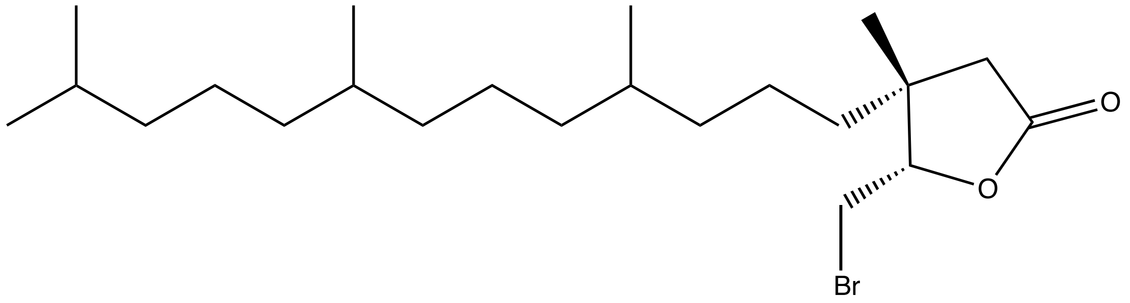


*trans*-5-bromomethyl-4-methyl-4-(4',8',12'-trimethyltridecyl)dihydrofuran-2-one **(4)**

**Figure S1: $^{1}H$ NMR spectrum of 4.**

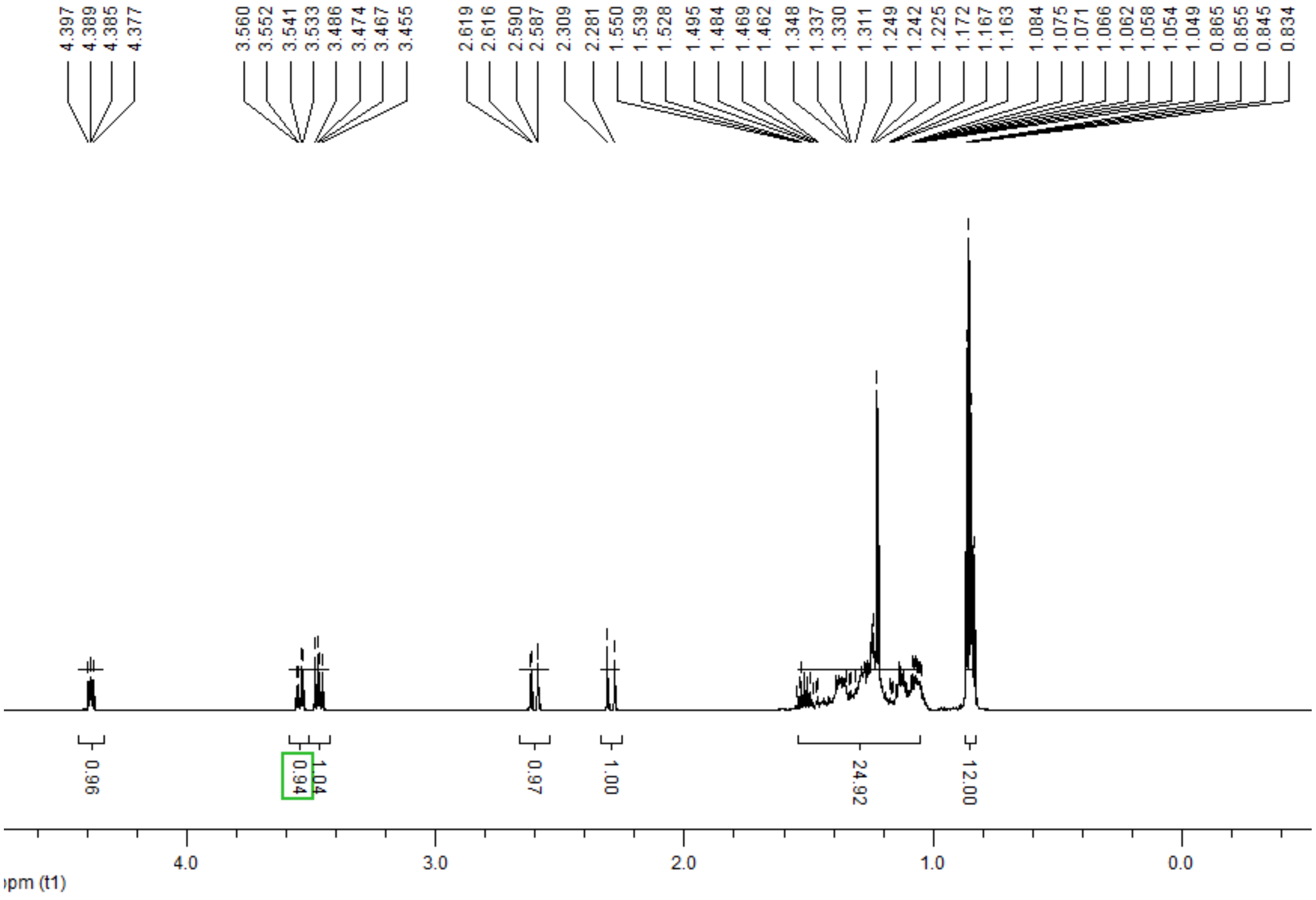

**Figure S2: $^{13}C$ NMR spectrum of 4.**

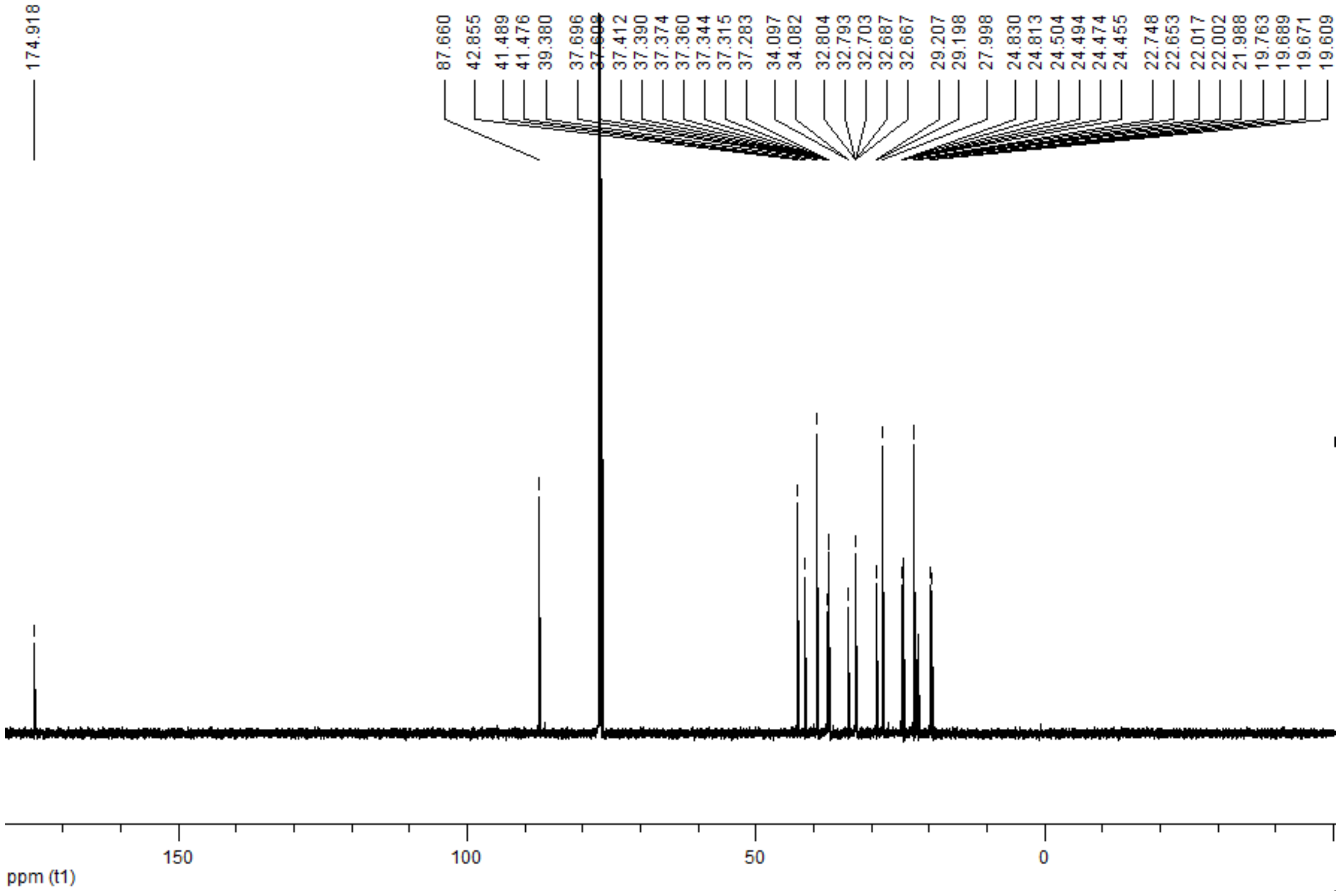

**Figure S3: $^{1}H$ – $^{1}H$ COSY spectrum of 4.**

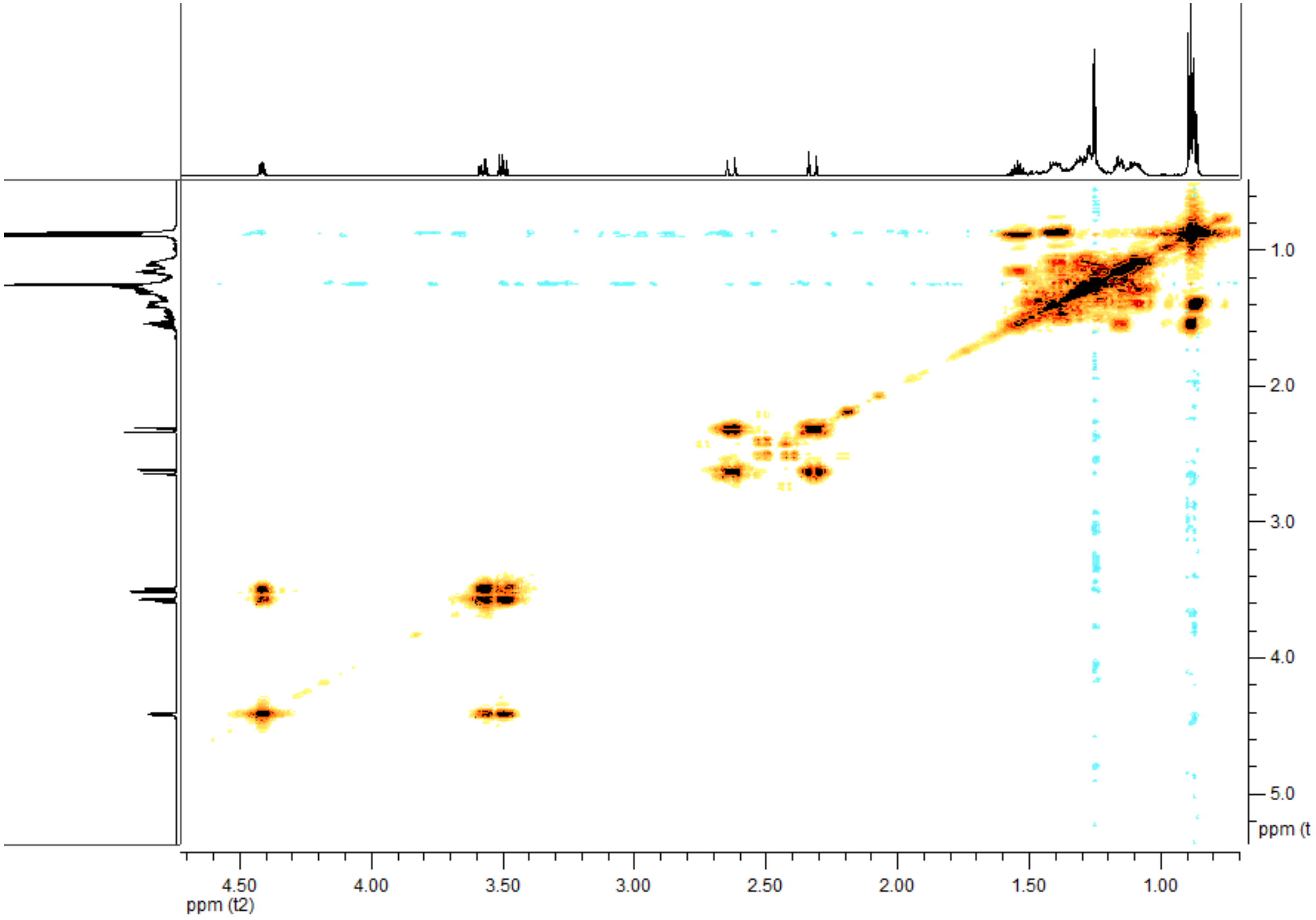

**Figure S4: HSQC spectrum of 4.**

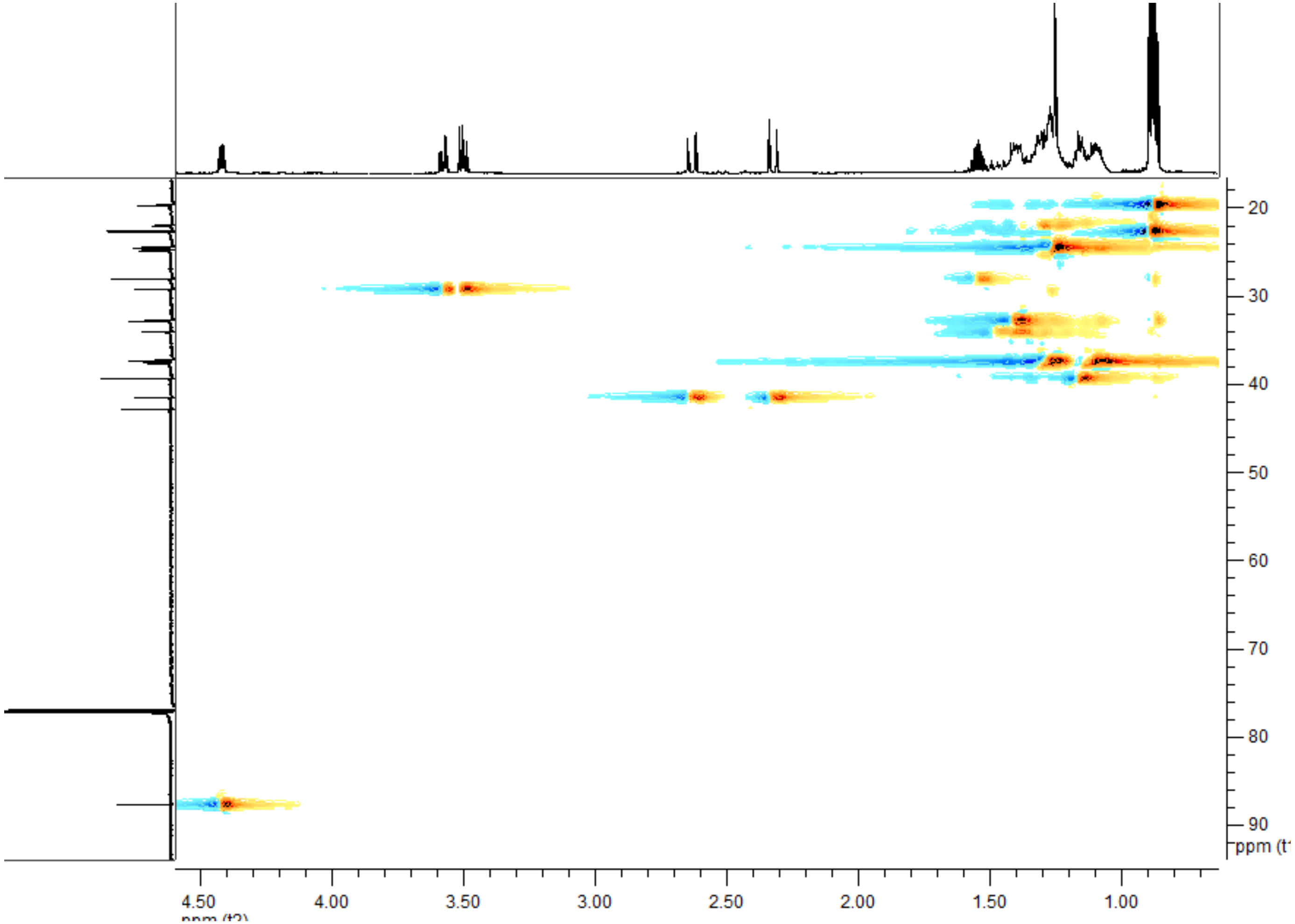

**Figure S5: NOESY spectrum of 4.**

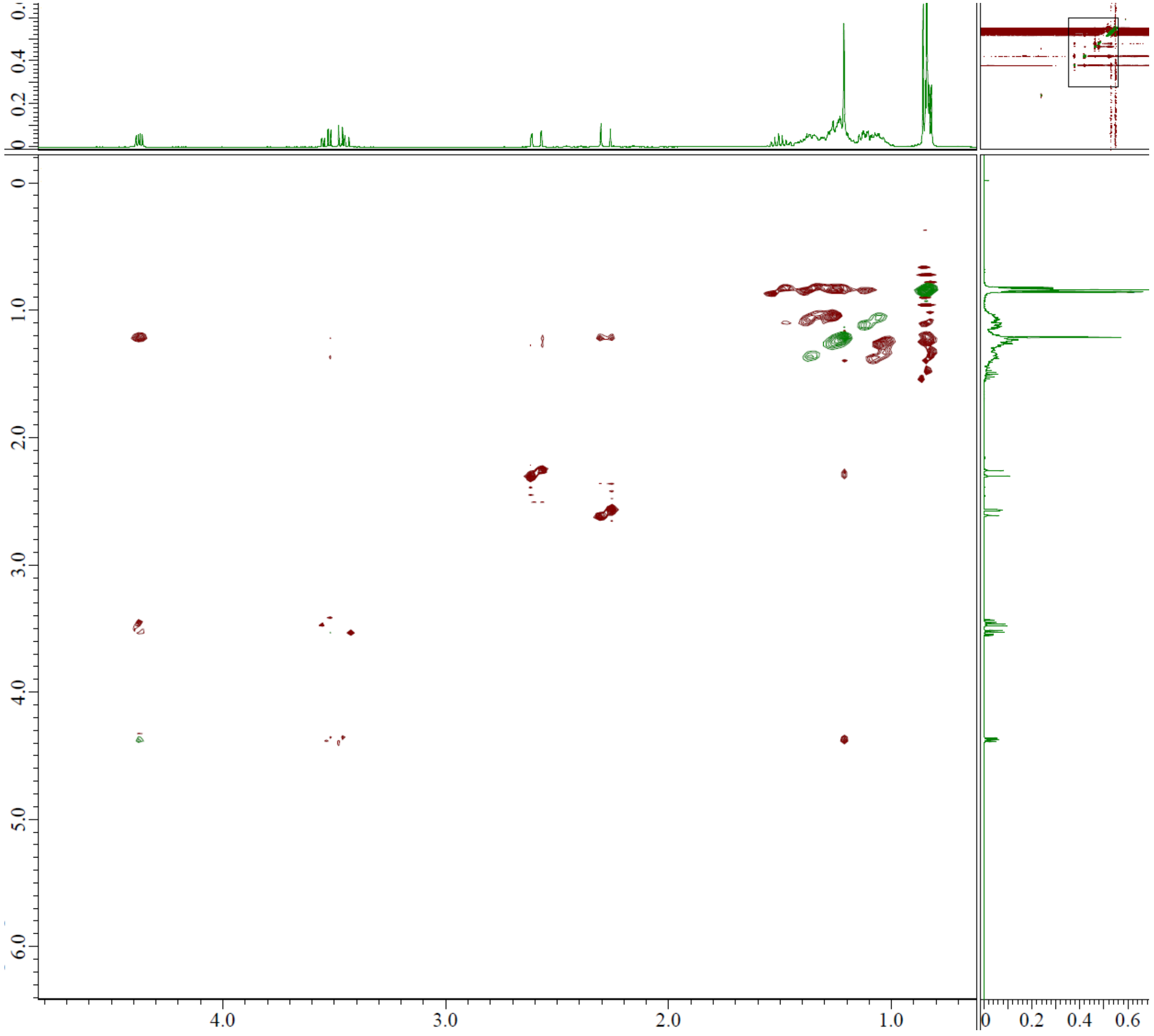

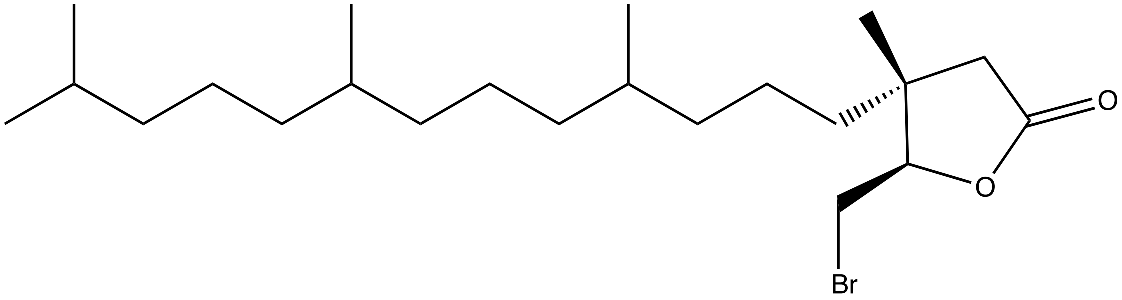


*cis*-5-bromomethyl-4-methyl-4-(4',8',12'-trimethyltridecyl)dihydrofuran-2-one (**5**)

**Figure S6: $^{1}$H NMR spectrum of 5.**

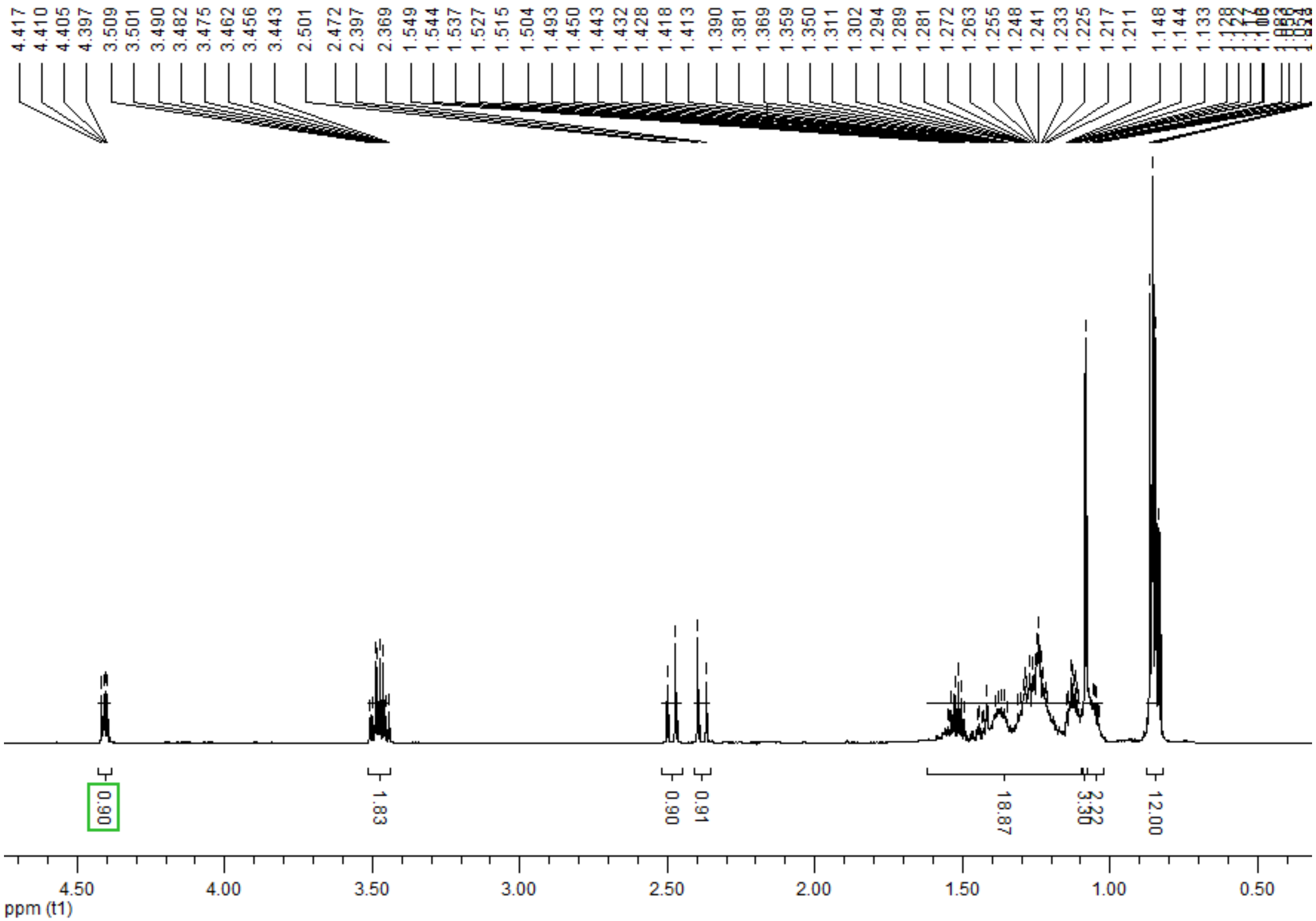

**Figure S7: $^{13}$C NMR spectrum of 5.**

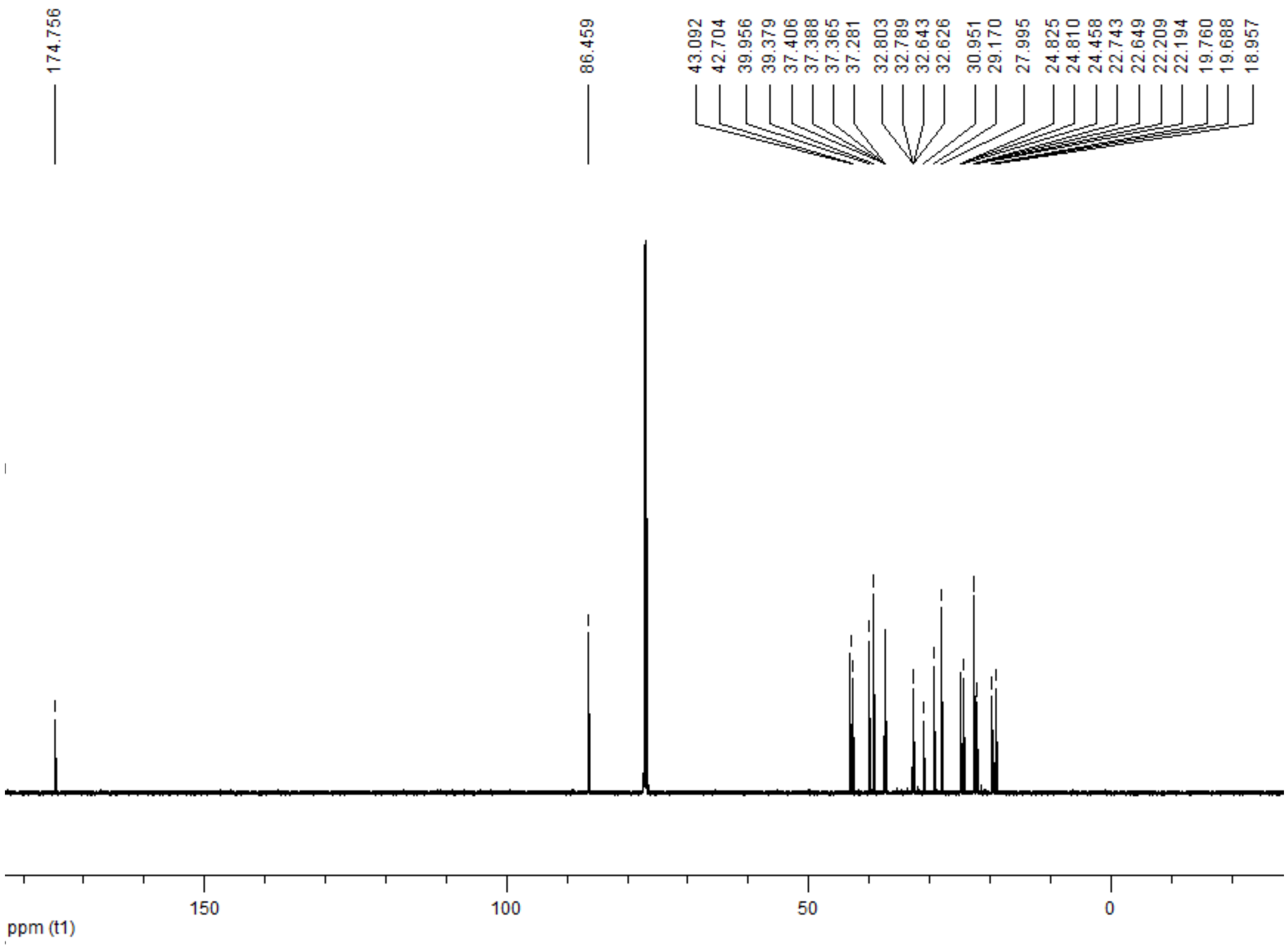

**Figure S8: $^{1}$H – $^{1}$H COSY spectrum of 5.**

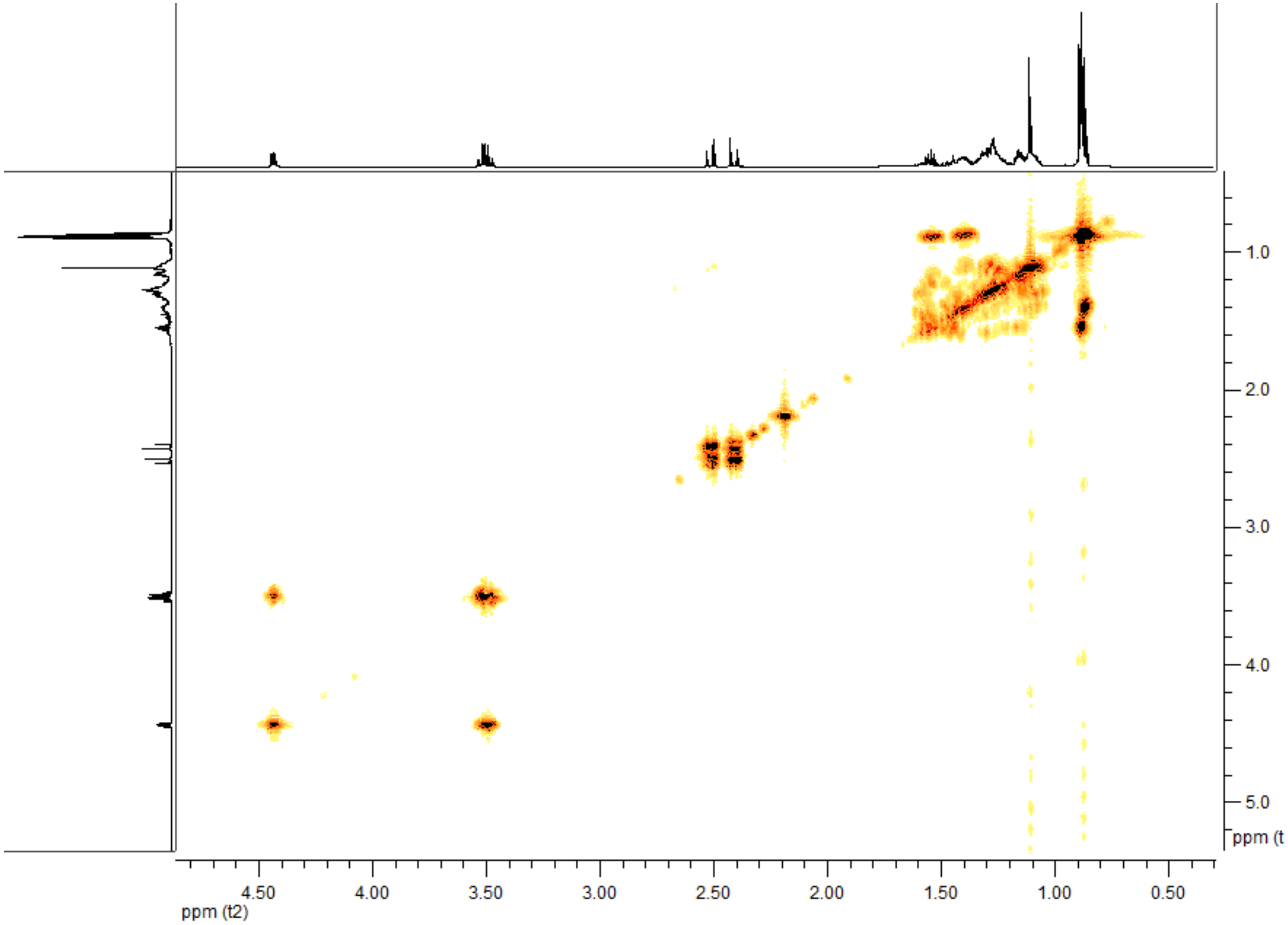

**Figure S9: HSQC spectrum of 5.**

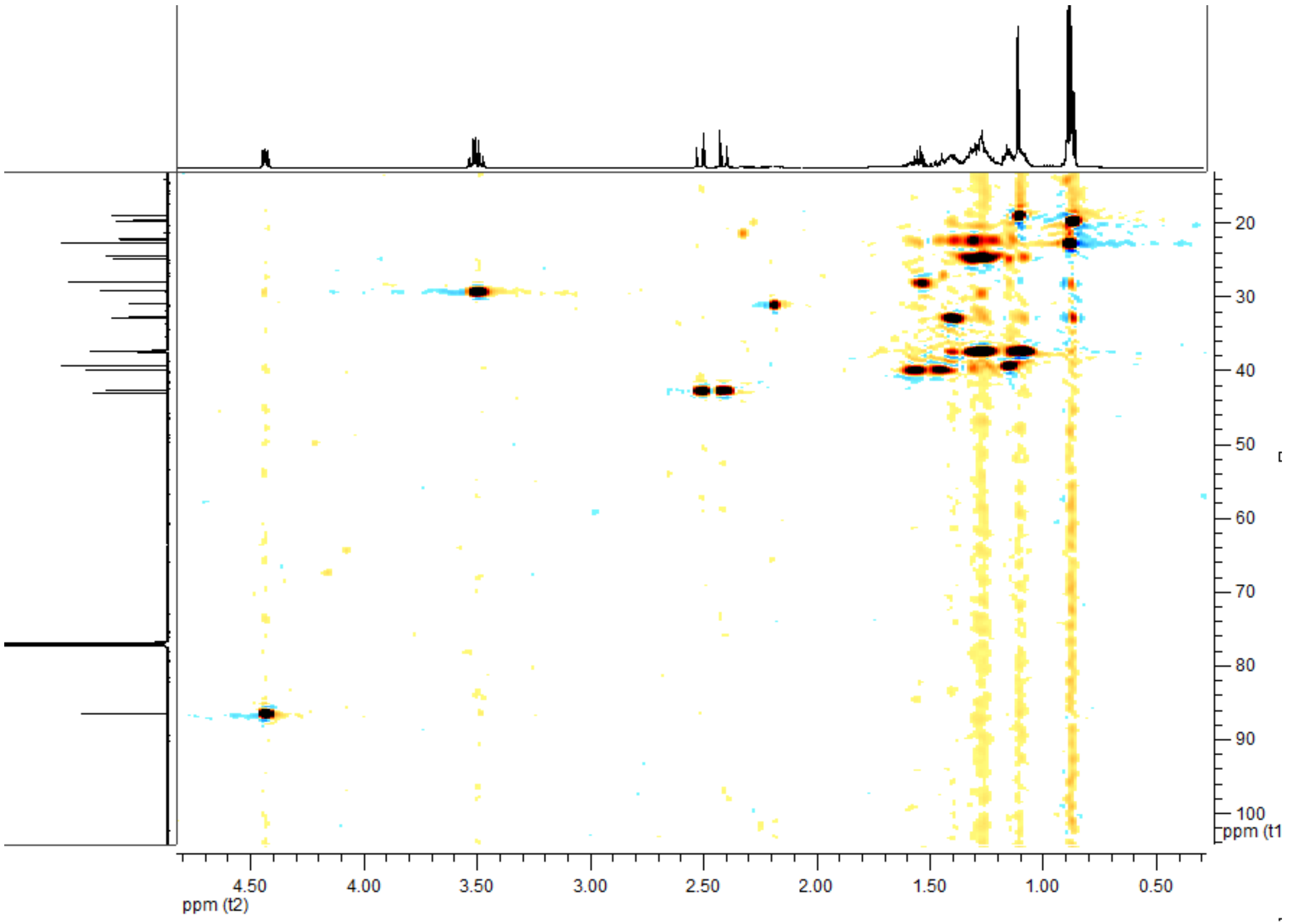

**Figure S10: NOESY spectrum of 5.**

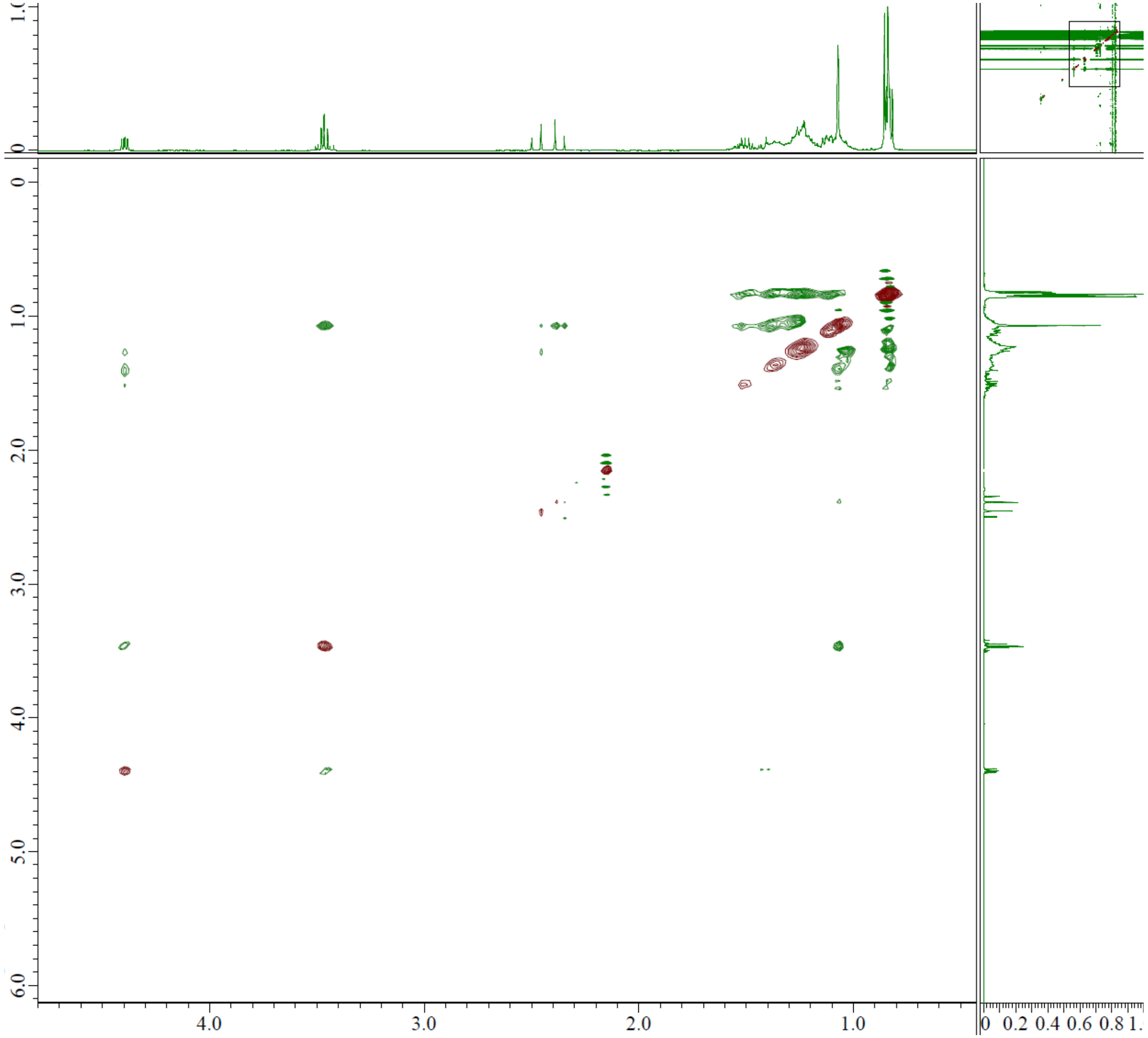

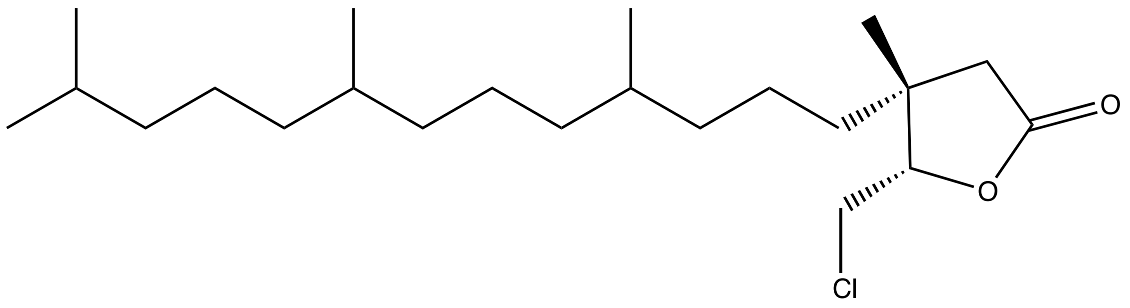


*trans*-5-chloromethyl-4-methyl-4-(4',8',12'-trimethyltridecyl)dihydrofuran-2-one **(6)**

**Figure S11: $^{1}$H NMR spectrum of 6.**

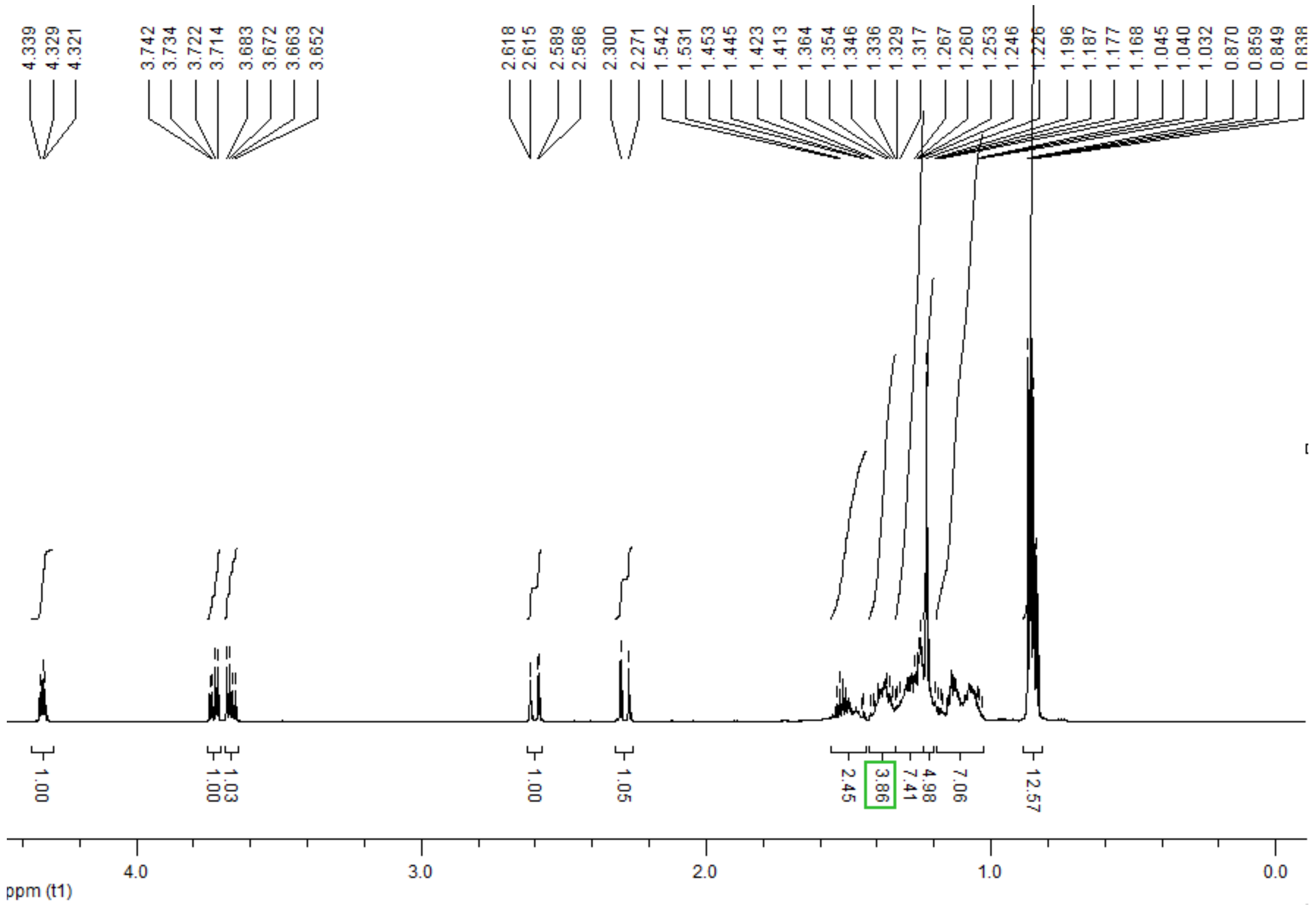

**Figure S12: $^{13}$C NMR spectrum of 6.**

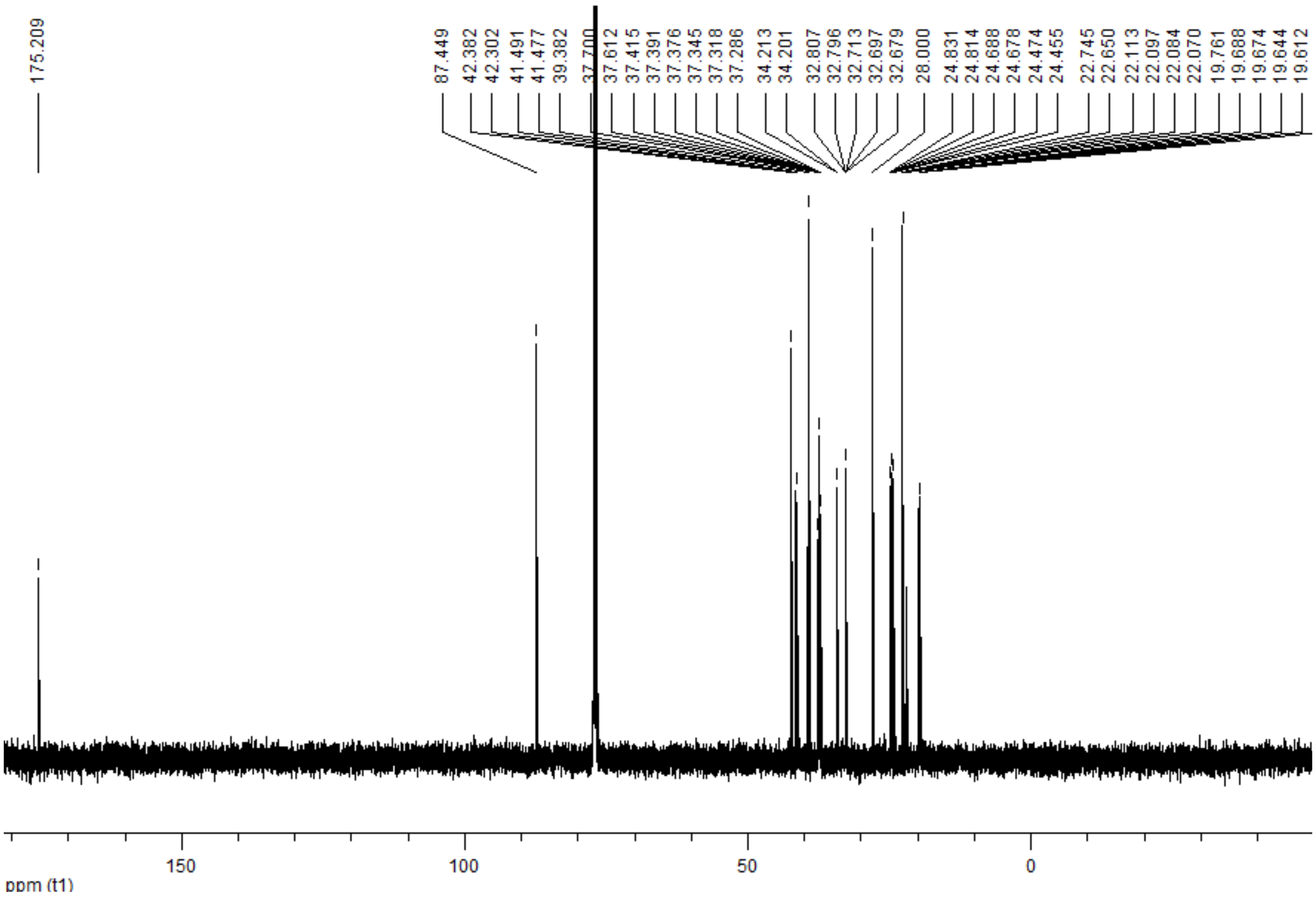

**Figure S13: $^{1}$H – $^{1}$H COSY spectrum of 6.**

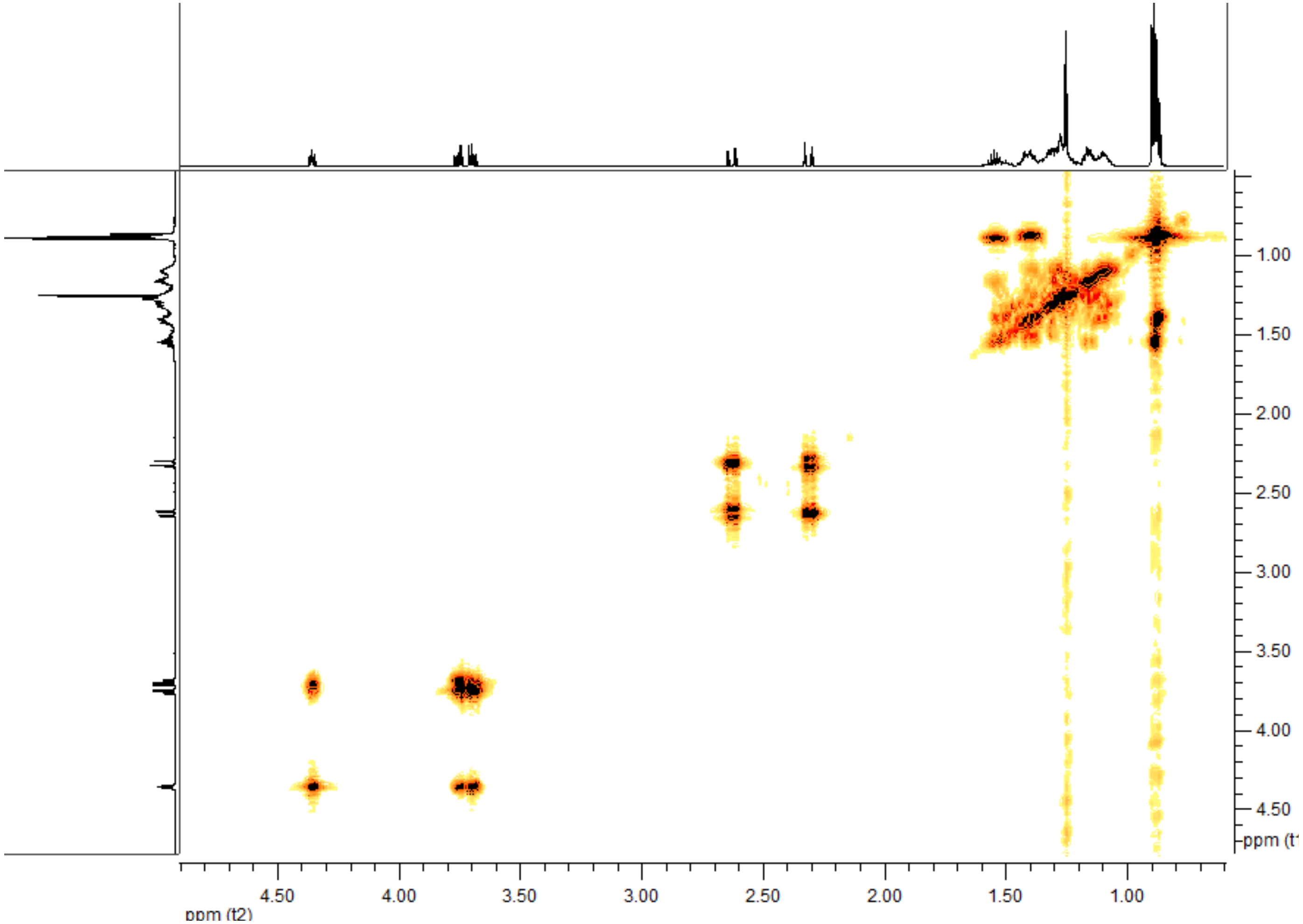

**Figure S14: HSQC spectrum of 6.**

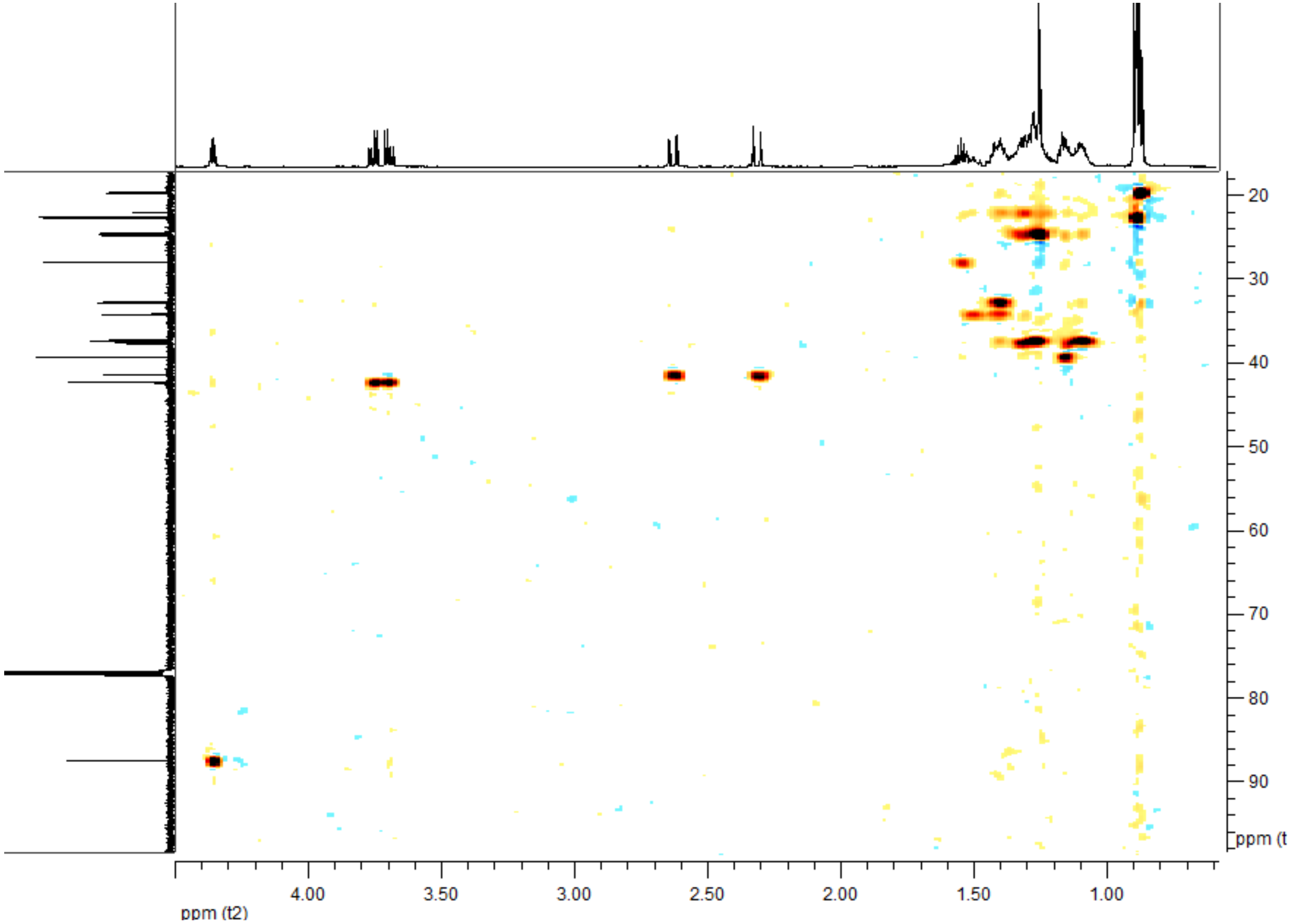

**Figure S15: NOESY spectrum of 6.**

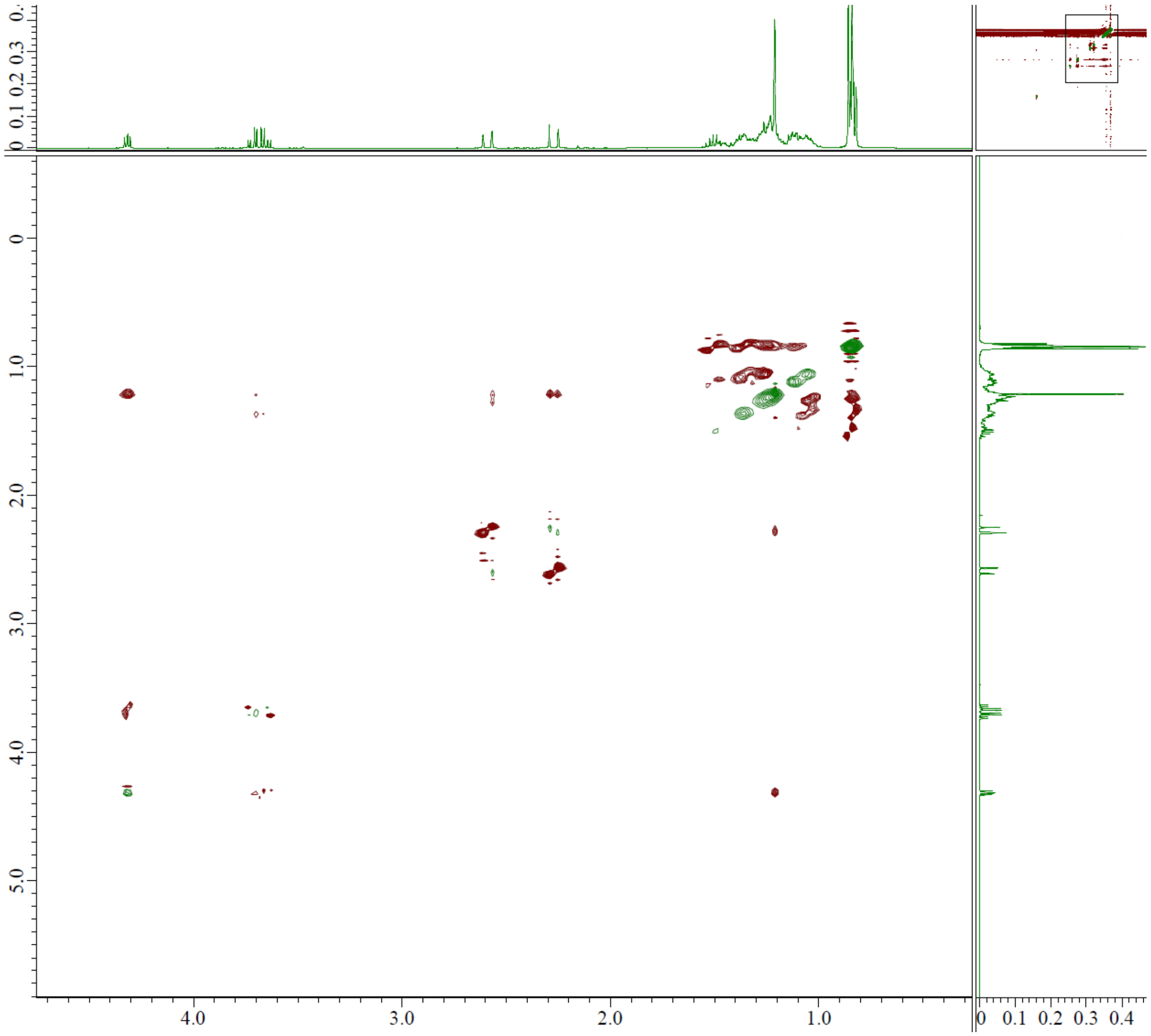

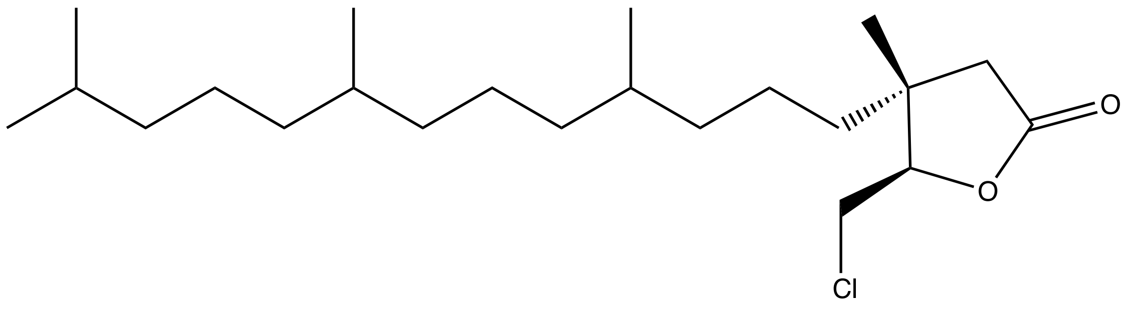


*cis*-5-chloromethyl-4-methyl-4-(4',8',12'-trimethyltridecyl)dihydrofuran-2-one (**7**)

**Figure S16: $^1$H NMR spectrum of 7.**

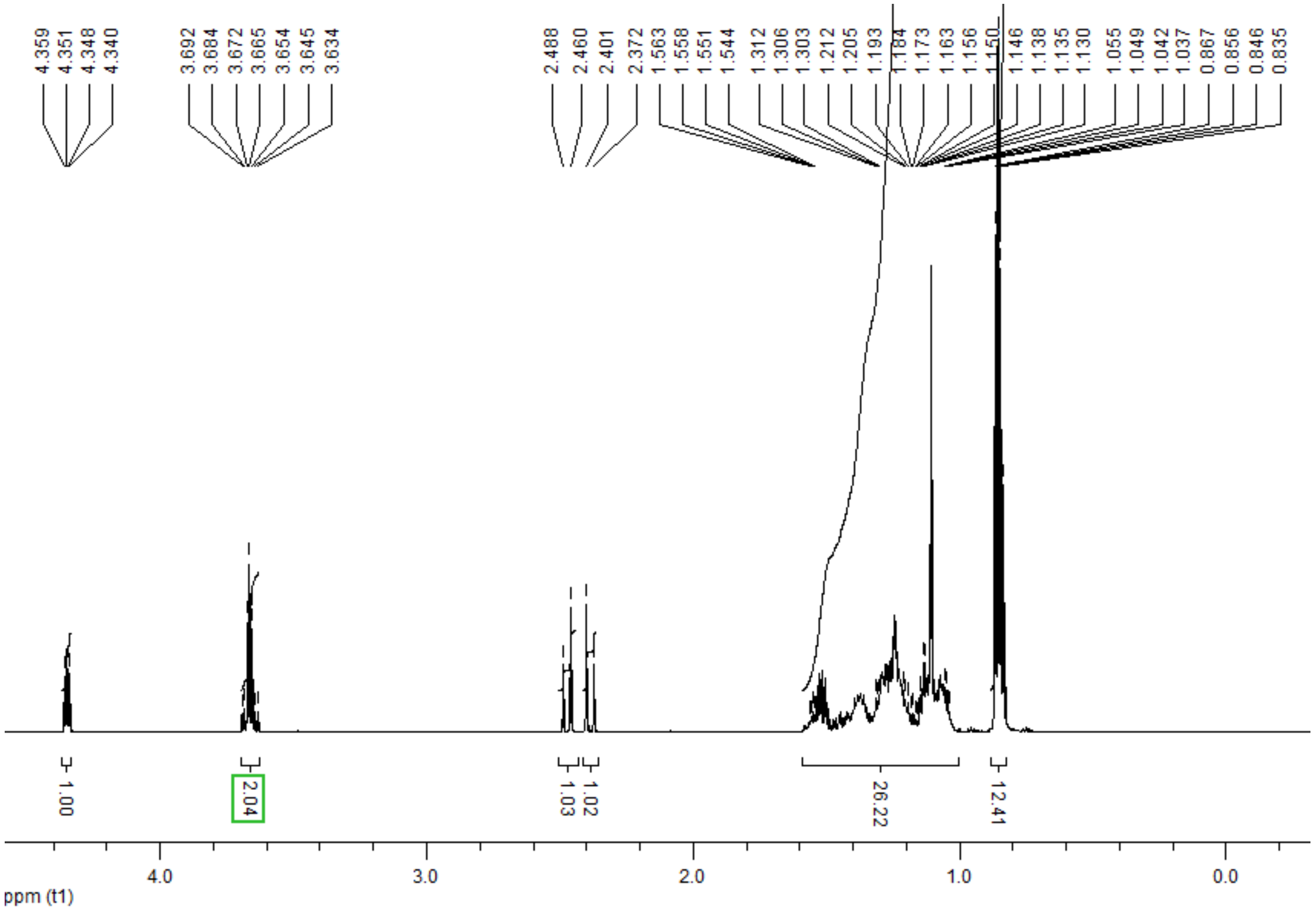

**Figure S17: $^{13}$C NMR spectrum of 7.**

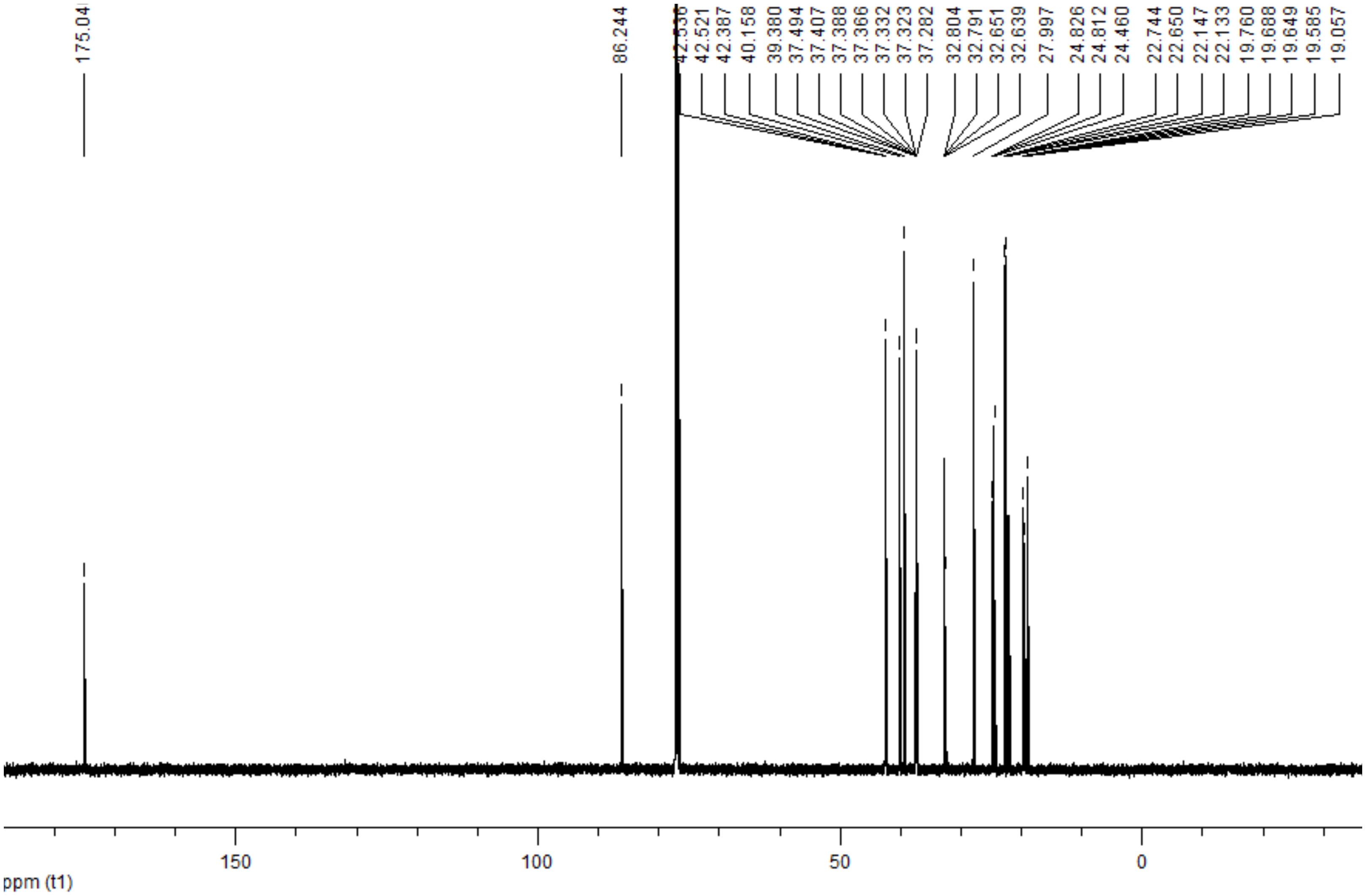

**Figure S18: $^1H - ^1H$ COSY spectrum of 7.**

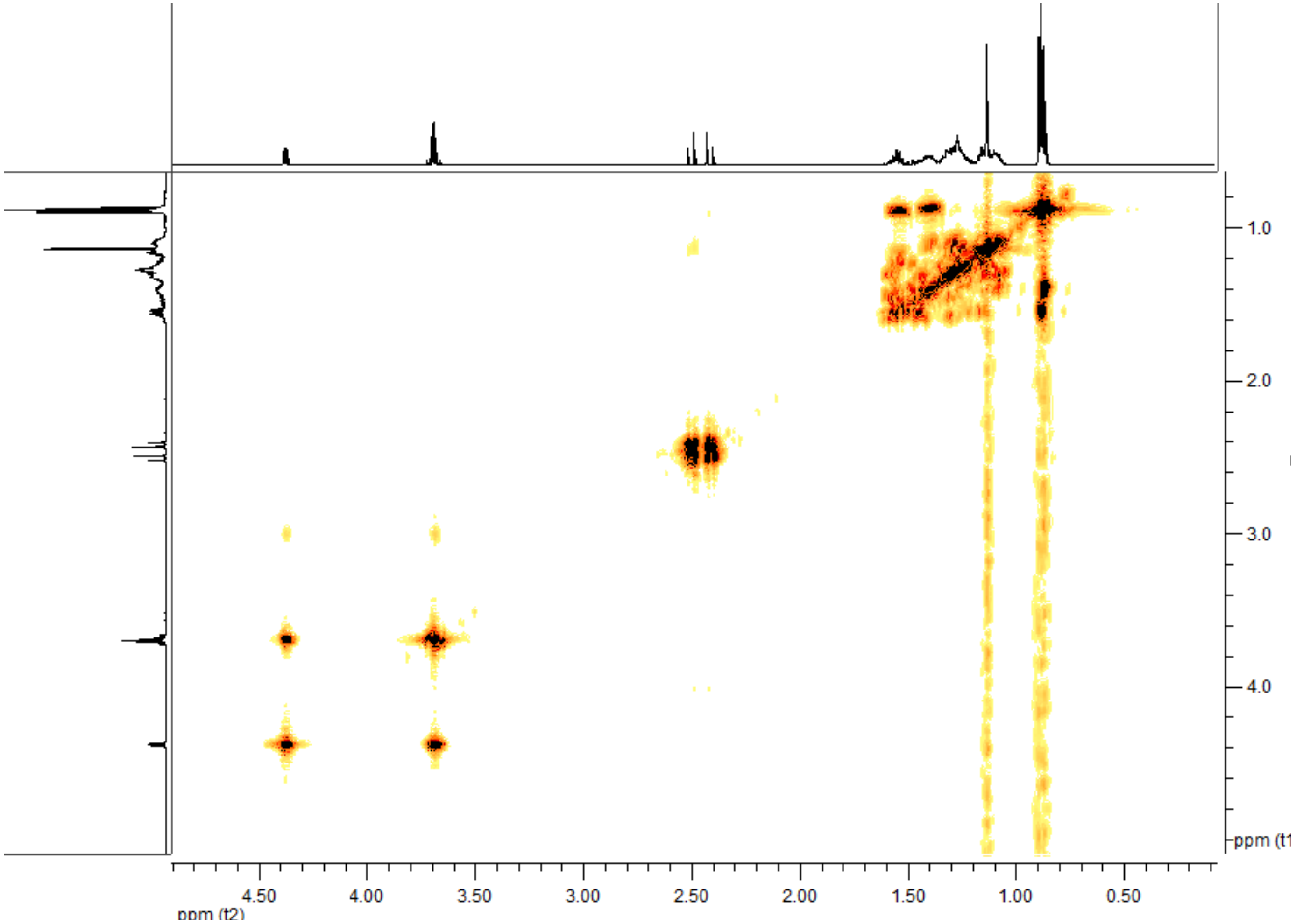

**Figure S19: HSQC spectrum of 7.**

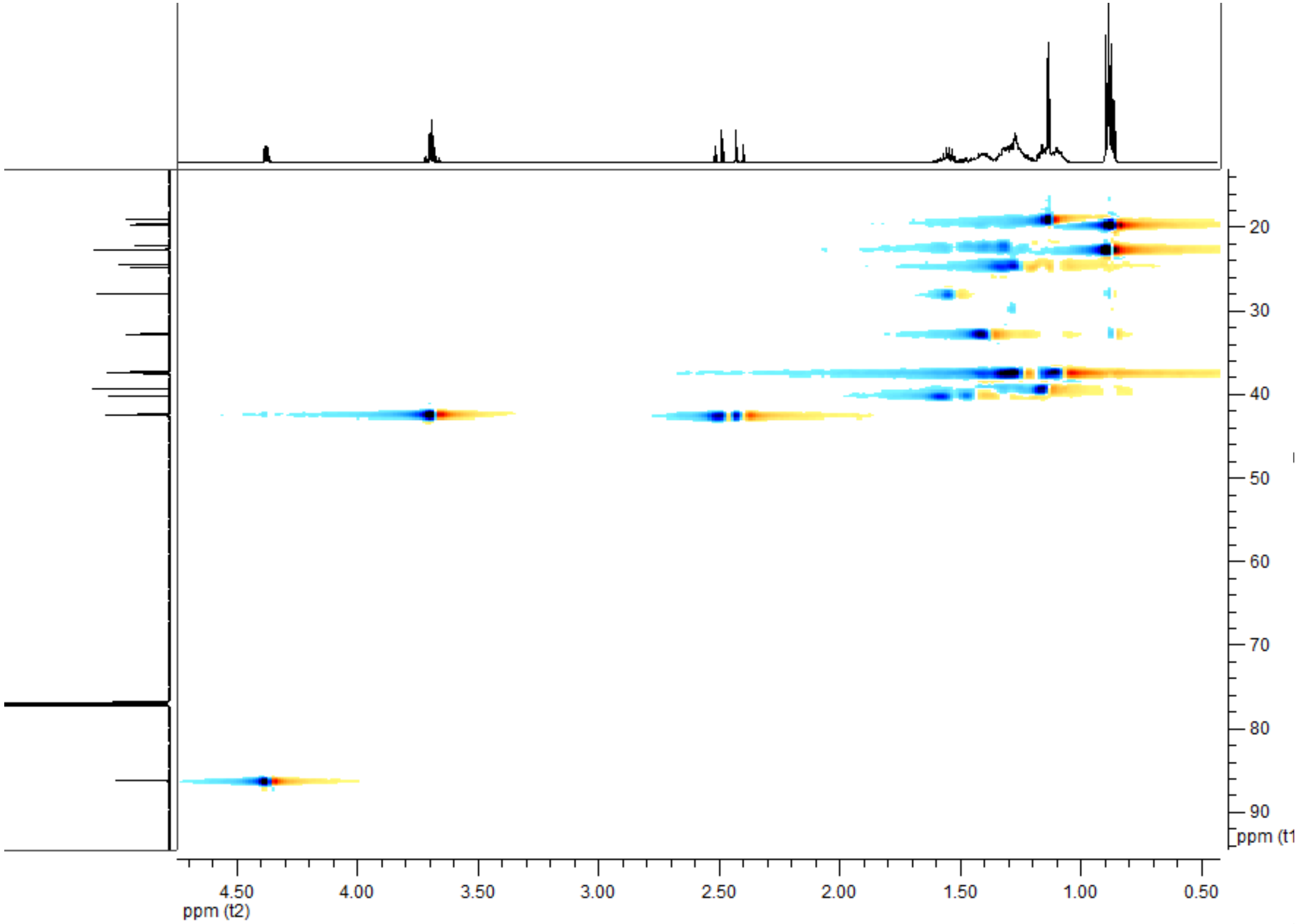

### Figure S20: NOESY spectrum of 7.

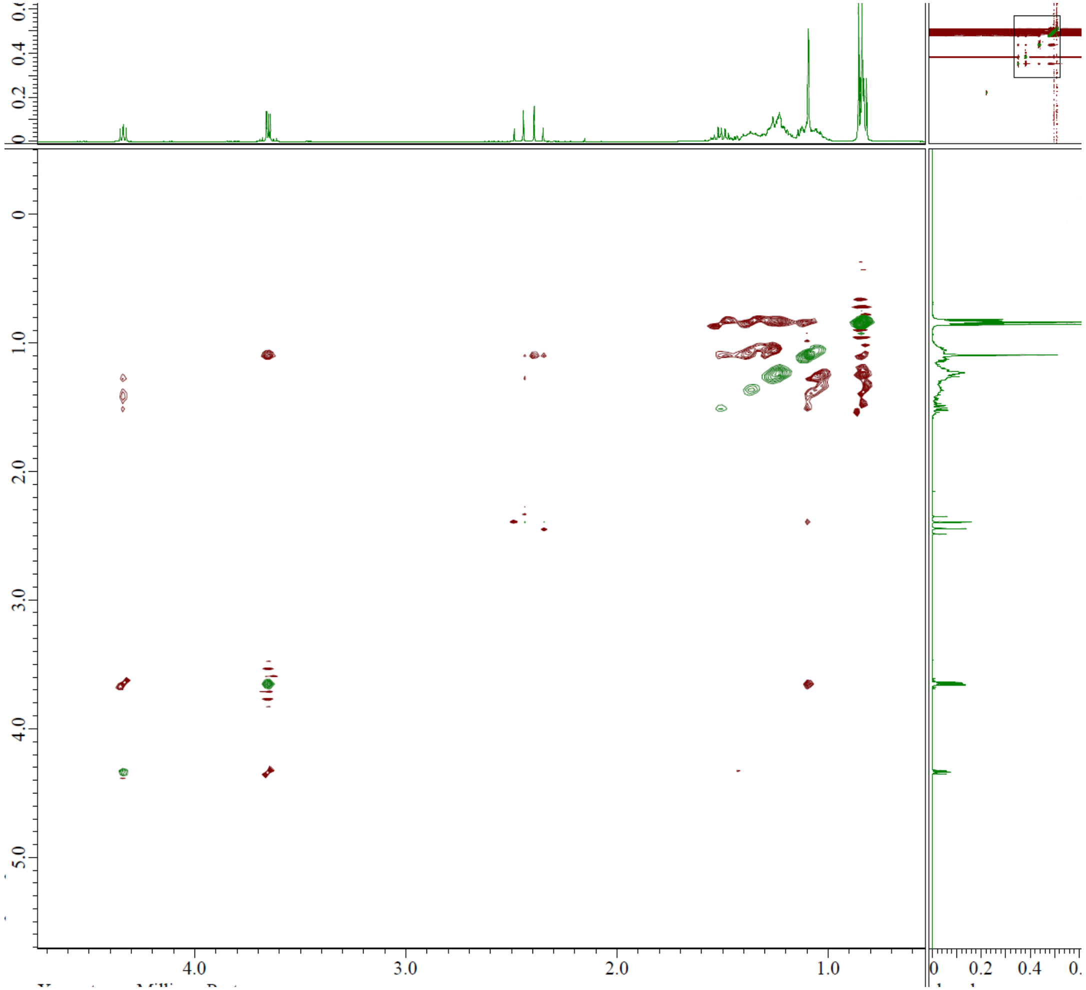

Supplement: Supplementary file 1 — Supplementary Information [file 41598_2021_83736_MOESM1_ESM.pdf]
